# Bacterial predator-prey coevolution accelerates genome evolution and selects on virulence-associated prey defences

**DOI:** 10.1038/s41467-019-12140-6

**Published:** 2019-09-20

**Authors:** Ramith R. Nair, Marie Vasse, Sébastien Wielgoss, Lei Sun, Yuen-Tsu N. Yu, Gregory J. Velicer

**Affiliations:** 1 Institute for Integrative Biology, ETH Zürich, Zürich, 8092 Switzerland; 2 Department of Systems Biology, Harvard Medical School, 02115 Boston, MA USA

**Keywords:** Microbial ecology, Coevolution, Experimental evolution, Molecular evolution

## Abstract

Generalist bacterial predators are likely to strongly shape many important ecological and evolutionary features of microbial communities, for example by altering the character and pace of molecular evolution, but investigations of such effects are scarce. Here we report how predator-prey interactions alter the evolution of fitness, genomes and phenotypic diversity in coevolving bacterial communities composed of *Myxococcus xanthus* as predator and *Escherichia coli* as prey, relative to single-species controls. We show evidence of reciprocal adaptation and demonstrate accelerated genomic evolution specific to coevolving communities, including the rapid appearance of mutator genotypes. Strong parallel evolution unique to the predator-prey communities occurs in both parties, with predators driving adaptation at two prey traits associated with virulence in bacterial pathogens—mucoidy and the outer-membrane protease OmpT. Our results suggest that generalist predatory bacteria are important determinants of how complex microbial communities and their interaction networks evolve in natural habitats.

## Abstract (web summary)

Predator-prey coevolution is expected to hasten evolutionary rates, but this is difficult to test in long-lived species. Here, the authors report consequences of experimental coevolution between bacterial predators and prey, including accelerated molecular evolution and parallel genomic and phenotypic adaptation.

## Introduction

Predation shapes communities and ecosystems in many ways, including by contributing to resource turnover^1,2^, driving the abundance and diversity of prey species^3,4^, inducing the evolution of novel predatory^5^ and prey-defense traits^6–8^ and indirectly altering interactions of prey with non-predatory species^3,9^. Sufficiently long periods of repeated interaction between predator and prey lineages can lead to Red Queen coevolution, in which cycles of reciprocal selection alter the biotic selective environment of both parties over time^10–13^. In turn, such increased variation of the biotic environment over time is expected to accelerate the pace of adaptive evolution^13–15^ relative to evolution in the absence of interaction.

Although predation is most often associated with animals, the microbial world is also rife with highly diverse predators. Protists consume a phylogenetically broad range of bacteria by ingesting whole cells^16^, with some being highly selective of prey cell size^17^. Bacterial predators have evolved both generalist and more specialized mechanisms of predation^18,19^. For example, myxobacteria such as *Myxococcus xanthus* are present across a broad range of microbial habitats^20^ and consume a wide diversity of prokaryotic and eukaryotic microbes^21–23^. The more specialized bacterial predator *Bdellovibrio bacteriovorus* consumes Gram-negative prey by using pili to invade the periplasm and subsequently feed internally on prey–cell contents before causing lysis and the release of predator offspring^24^. Bacteriophages are obligate parasites (also often referred to as predators) that inject their genetic material into host cells via attachment to specific cell-surface molecules and often have narrow host ranges^25^. Previous evolution experiments have shown that prey evolution can be influenced by predation from protists^26^, specialized bacterial predators^27^ and phage^28^, and that the spatial distribution of prey can determine how bacterial predatory behaviors evolve^29^. However, to our knowledge, it remains largely unexplored how predation by generalist bacterial predators shapes the genomic and phenotypic evolution of coevolving predator–prey communities, as well as the evolution of interaction networks within complex microbial communities.

*Myxococcus xanthus* is perhaps best known for its cooperative formation of multicellular fruiting bodies upon starvation^20^, but as a predator *M. xanthus* consumes a wide variety of other microbial species with varying degrees of efficiency^22,23^. For example, *M. xanthus* predates efficiently on *Escherichia coli*^23^, a human-gut symbiont and occasional pathogen of the digestive and urinary tracts and bloodstream^30^ that can also be isolated from non-host environments including soil^31^. The precise molecular mechanisms by which *M. xanthus* cells externally lyse and decompose prey cells remain poorly understood^21,32^, but can involve secreted antibiotics and extracellular digestive enzymes, some of which may be released from outer-membrane vesicles^21,33^. It has been proposed that, like fruiting-body development^34^, predatory killing and lysis of prey cells by *M. xanthus* involves density-dependent cooperation (and has thus been likened to “wolf-pack” predation)^35^, but evidence offered in support of this hypothesis is indirect^32,36^.

**Fig. 1:**
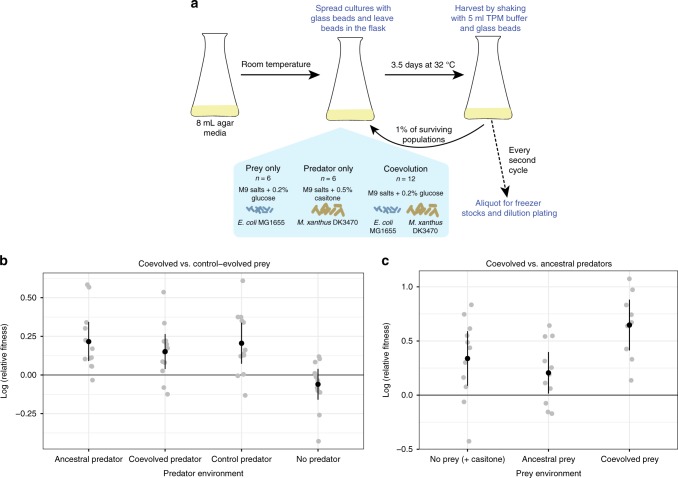
Fitness patterns indicate adaptation specific to the coevolution treatment. **a** Coevolved predator-prey communities (12) and control-evolved predator or prey populations (six each) were propagated on minimal medium supplemented with glucose or casitone. The figure was created by the authors. **b** Fitness of coevolved prey relative to control-evolved prey in direct competition experiments in the presence and absence of predators (*n* = 12). **c** Fitness of coevolved relative to ancestral predators in direct competitions in the presence of prey and during growth on casitone in the absence of prey (*n* = 12). Gray dots are population means, black dots are treatment means and error bars show 95% confidence intervals (*t*-distribution). Source data are provided as a Source Data file

Here, we test whether and how interaction between *M. xanthus* as predator and *E. coli* as prey in co-evolving two-species communities alters genomic and phenotypic evolution relative to single-species populations evolving under the same (or similar) abiotic conditions. To do so, we establish communities in which *M. xanthus* is trophically dependent on *E. coli* as its sole direct carbon substrate for growth, while the prey grows on an added monosaccharide (glucose) that *M. xanthus* is unable to utilize. Glucose is the sole abiotic carbon source provided in the coevolution treatment, in the prey-only control treatment and in one of two predator-only control treatments (Fig. 1a, Supplementary Table 1). Because *M. xanthus* is predicted to go extinct in the predator-only control with glucose minimal medium, we establish a second predator-only control using the same basal buffered medium as the other treatments but supplemented with casitone, an amino-acid-rich carbon source allowing predator growth in the absence of prey. Replicate communities and populations undergo 25 growth cycles (3.5 days each, see Methods). At the end of each cycle, 1% of each community or population is randomly transferred to a fresh media flask, giving ~6.64 generations of replacement growth per cycle, or ~166 generations over the entire experiment. However, generation numbers may be somewhat increased for prey in coevolving populations due to predation.

Red Queen coevolution is expected to occur over extended periods in this arena. In this initial time-limited experiment, we find fitness patterns among the coevolved prey and predator lineages indicative of reciprocal adaptation. In addition, coevolved lineages of both predators and prey evolve faster, i.e., they accumulate more mutations, compared to control lineages evolved in isolation. We further identify strong signatures of parallel evolution at both genomic and phenotypic levels specific to the predator–prey communities, highlighting the importance of the biotic selective pressure in shaping the evolution of both parties.

## Results

### Reciprocal adaptation in predator–prey communities

As expected, all predator-only populations inoculated on glucose-minimal medium rapidly went extinct (within five cycles). All other populations of both predator and prey persisted for the duration of the experiment. Adaptation was quantified with pairwise competitions between coevolved vs. control-evolved prey in the presence and absence of predators (ancestral, coevolved, and control-evolved) and between coevolved vs. ancestral predators in the presence and absence of prey (ancestral and coevolved). We modified an existing NGS-based method for measuring frequencies of genetically distinct bacterial competitors (FreqSeq^37^) to simultaneously estimate competitor frequencies from hundreds of competition experiments (Multiplex FreqSeq (Supplementary Fig. 1, Supplementary Tables 2 and 3)). Control experiments showed Multiplex FreqSeq to estimate *E. coli* competitor frequencies with high accuracy (Supplementary Fig. 2).

In direct competitions between coevolved vs. control-evolved prey, the coevolved populations were fitter in all three predator contexts but not in the absence of predators, thereby indicating adaptive evolution of prey in response to general predation pressure (ANOVA, predator treatment: *F*_3,128_ = 22.07, *p* = 1.4 × 10^−11^, one-sample *t* tests with Holm–Bonferroni correction; ancestral: *t*_11_ = 3.78, *p* = 0.012; coevolved: *t*_11_ = 2.95, *p* = 0.027; control-evolved: *t*_11_ = 3.42, *p* = 0.017; no predator: *t*_11_ = −1.32, *p* = 0.21; Fig. 1b, Supplementary Fig. 3). On the predator side, coevolved predators outcompeted their ancestors in all tested environments (including in the absence of prey; ANOVA, prey treatment: *F*_2,57_ = 4.908, *p* = 0.0115; one-sample *t* tests with Holm–Bonferroni correction; coevolved prey: *t*_8_ = 6.38, *p* = 6.4 × 10^−4^; ancestral prey: *t*_10_ = 2.39, *p* = 0.038; casitone: *t*_10_ = 2.99, *p* = 0.027, Fig. 1c, Supplementary Fig. 4), but did so to a greater degree while consuming coevolved prey than while consuming ancestral prey or casitone (Tukey multiple comparisons of means, coevolved vs. ancestral *p* = 0.047, coevolved vs. casitone *p* = 0.011). Although the prey vs. predator fitness competitions differ in design and are thus not directly comparable, their collective outcomes suggest that predator vs. prey adaptation may differ in patterns of specificity across evolutionary stages.

### Parallel evolution of mucoidy among prey populations

Striking patterns of parallel evolution—both phenotypic and genomic—were detected uniquely in the predator–prey coevolution treatment. At the phenotypic level, most coevolved prey populations contained readily detectable frequencies of mucoid-colony forming variants during the experiment. For example, at cycle 18, mucoids were found in ten of the twelve coevolved populations (at frequencies ranging from ~0.0008 to ~0.2), whereas mucoidy was detected in only one prey-only control population (and there in only one colony, Supplementary Table 4). This nearly complete restriction of mucoid genotypes to predator–prey communities is extremely unlikely to have occurred by chance (exact binomial test, *p* < 0.001), strongly indicating that predation imposes positive selection for mucoidy, a known virulence trait in *E. coli*^38^ that also reduces susceptibility to phage infection in other species^39^.

**Fig. 2:**
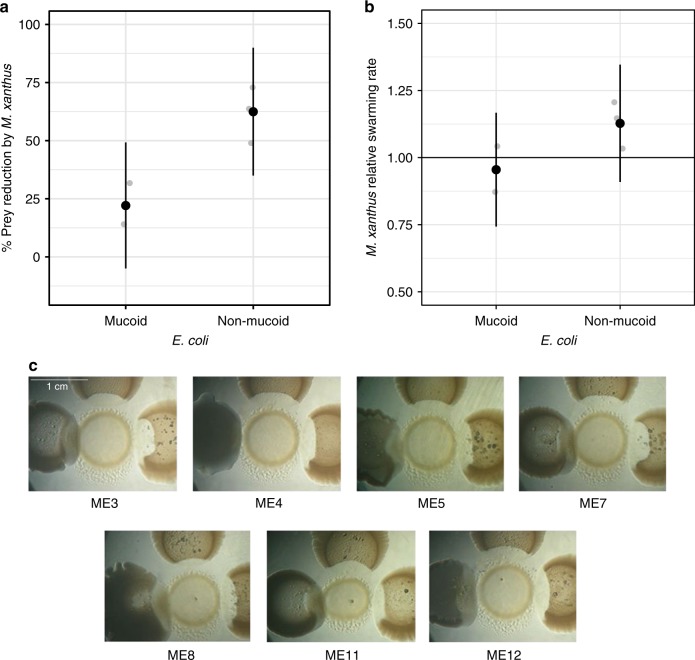
Mucoidy is associated with reduced predator efficacy among coevolved prey. **a** End-of-cycle non-mucoid populations are reduced more by predation than mucoid populations (*n* = 3). **b** Swarming speed (relative to speed on ancestral prey) of the ancestral predator is slower through lawns of a coevolved mucoid strain than a non-mucoid strain (*n* = 3). Gray dots are individual replicates, black dots are means and error bars show 95% confidence intervals (*t*-distribution). **c** Qualitative predation assay of *M. xanthus* (center) on mucoid (left), non-mucoid (right), and ancestral (above) *E. coli* from seven coevolved populations. Source data are provided as a Source Data file

Multiple results point to predation-specific advantages of mucoidy. After a cycle of growth and predation under the same conditions as in the co-evolution experiment, the population sizes of both a non-mucoid and a mucoid isolate from the same co-evolved *E. coli* population (ME4) were reduced by *M. xanthus* relative to predator-free cultures (Fig. 2a). However, this negative effect of the predator was significantly lower for the mucoid strain (one-sided *t* test: *t*_4_ = −4.497, *p* = 0.005; Fig. 2a).

Mucoidy was also associated with slower swarming of *M. xanthus* colonies through prey lawns, a parameter previously found to correlate with predatory killing and other measures of predatory performance across a broad range of prey types^23^. Colonies of our ancestral predator genotype swarmed more slowly through a lawn of the coevolved ME4 mucoid *E. coli* strain than through the ME4 non-mucoid strain (one-sided *t* test: *t*_4_ = −2.4386, *p* = 0.036, Fig. 2b). More broadly, we observed a generally reduced ability of *M. xanthus* to penetrate, dissolve and swarm through mucoid colonies relative to non-mucoid colonies across isolates from different replicate co-evolved communities (Fig. 2c). Collectively, our results suggest that secreted polymers causing the mucoid phenotype decrease susceptibility to predation, perhaps simply by hindering access of predator molecules to the prey cell wall.

### Parallel genome evolution among both prey and predators

**Fig. 3:**
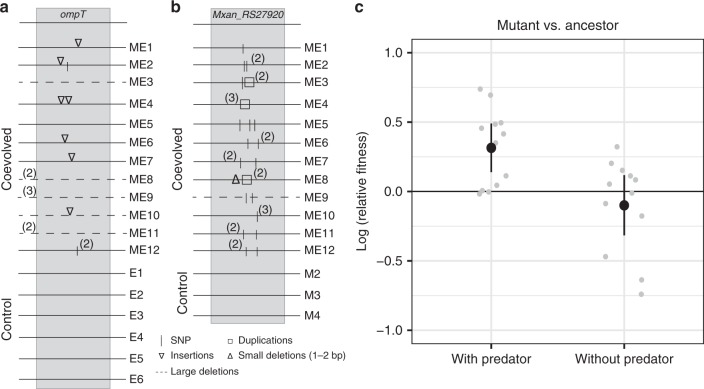
Parallel genomic evolution reveals adaptation specific to the coevolution treatment. **a** Parallel mutation of *ompT* among coevolved prey populations. **b** Parallel mutation of *Mxan_RS27920* among coevolved predator populations. **a**, **b** Numbers in parentheses indicate the number of sampled clones (of three) in a population sharing the adjacent mutation. **c** Fitness of an *ompT*-deletion mutant in competition with the *ompT*^*+*^ ancestor in the presence and absence of the ancestral predator (*n* *=* 6 for both treatments). Gray dots are individual data points, black dots are means and error bars represent 95% confidence intervals (*t*-distribution). Source data are provided as a Source Data file

To investigate the impact of bacterial predator–prey coevolution on the genomic evolution of both predatory antagonists and victims, we sequenced the genomes of three clones from each coevolved and control-evolved population of predators and prey. Concomitant with parallel evolution of mucoid phenotypes among prey, coevolved populations of both predators and prey exhibited striking patterns of parallel genotypic evolution that control populations did not. Multiple loci among both prey and predator coevolved populations evolved in parallel (Fig. 3a, b; Supplementary Tables 5–8), including extreme parallelism at *ompT* (Fig. 3a) among prey and an uncharacterized locus (*Mxan_RS27920*) among predators (Fig. 3b).

The gene *ompT*, which encodes an outer-membrane protease^40^, was mutated in 11 out of the 12 coevolved prey populations (19 clones out of 36), with no individual mutation shared by clones from distinct populations. In contrast, this gene was not mutated in any of the six control-evolved populations (Fig. 3a). Many *ompT* mutations were multi-base deletions or generated premature stop codons, indicating predation-specific selection against *ompT* function. As predicted from the mutational patterns among evolved clones, experimental deletion of *ompT* in the ancestral *E. coli* genetic background conferred a significant fitness advantage when competitions were performed with predation pressure from *M. xanthus* but not in the absence of the predator (ANOVA, predator treatment: *F*_1,16_ = 8.42, *p* = 0.01, one-sample *t* tests with Holm–Bonferroni correction; presence: *t*_11_ = 3.96, *p* = 0.005 and absence: *t*_11_ = −1.006, *p* = 0.34; Fig. 3c, Supplementary Fig. 5).

Among the predators, the *Mxan_RS27920* locus was mutated or deleted in almost all clones from the 12 coevolved populations (34/36) but in none of the clones from casitone-evolved lineages, again with no individual mutations shared across populations.

### Accelerated genome evolution in predator–prey communities

**Fig. 4:**
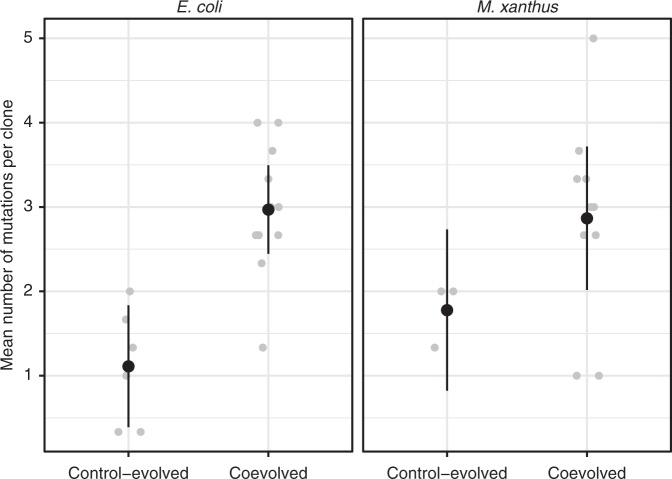
Predator–prey coevolution accelerates genome evolution. Significantly more mutations were present among clones from coevolved prey and predator populations than in control populations (*n* = 3 clones for each species for most individual populations, total *n* = 33 and *n* = 18 for coevolved and control-evolved prey populations, respectively; *n* = 31 and *n* = 8 for coevolved and casitone-evolved predator populations, respectively). Mutator clones (three clones from one coevolved prey population and five clones from two coevolved predator populations) were excluded. Gray dots are the means across the included clones for each population, black dots are treatment means and error bars represent 95% confidence intervals (*t*-distribution) of treatment means. Source data are provided as a Source Data file

We tested for differences in the rate of genomic evolution between the coevolution vs. control treatments for both predators and prey, as measured by the average number of mutations present in evolved clones at the end of cycle 25. Even excluding mutator clones with elevated mutation rates^41^ (which appeared only in the coevolution treatment), prey that evolved under predation pressure accumulated ~2.7-fold more mutations on average than control-evolved prey clones (one-sided *t* test: *t*_11_ = 5.07, *p* = 0.0002, Fig. 4). Again, excluding mutators, genomic evolution was also accelerated among predators (~1.6-fold) in the coevolution treatment relative to control populations growing on casitone (one-sided *t* test: *t*_11_ = 2.49, *p* = 0.015, Fig. 4). Predator–prey interactions may be responsible for this effect, but due to supplementation of media with casitone rather than glucose in this treatment (to allow predator survival) the possibility of differential abiotic carbon-source effects on predator genome evolution cannot be excluded.

High frequencies of mutator clones with elevated numbers of mutations were found in three of the twelve coevolved communities, once on the prey side and twice on the predator side. The three *E. coli* clones sampled from ME1 carry 15–23 mutations per clone and share a mutation in the mismatch repair gene *mutL* (Supplementary Table 5). The three *M. xanthus* clones sampled from ME4 carry 96–109 mutations, including shared mutations in the DNA repair genes *mutS* and *recN* (Supplementary Table 6) and two *M. xanthus* clones from ME8 (43 and 45 mutations total) also carry a *mutS* mutation (Supplementary Table 6). Overall, our results clearly show that bacterial predator-prey interactions increase genome evolution among prey, suggest the same for predators and also suggest that predation may promote the evolution of elevated mutation rates.

## Discussion

In this study, fitness patterns indicate adaptation by both predators and prey specific to the coevolution treatment suggestive of early stages of Red Queen coevolution. In direct competition experiments between coevolved vs. control prey, the coevolved populations collectively exhibited higher fitness than the controls in the presence of all three predator types (ancestral, control-evolved, and co-evolved) but not in the absence of predation (Fig. 1b). These results suggest that coevolved prey adapted in response to traits shared by all predator types. In contrast, while coevolved predators appear to be more fit than their ancestors in all three examined environments, they have a significantly greater fitness advantage while growing on co-evolved prey than on ancestral prey (or on casitone in the absence of prey) (Fig. 1c). This pattern suggests that the predators may have adapted in response to earlier-evolved prey adaptations.

Antagonistic coevolution is predicted to increase the pace of adaptive molecular evolution and this has been observed among rapidly coevolving phage^15^. However, such increased evolution has not to our knowledge been detected among prey or other predator types with slower generation times than phage^15^. In this study, genomic evolution was found to be greatly accelerated among both prey and predators in the two-species coevolution treatment compared to the respective single-species control populations. Even excluding co-evolved mutator clones, nearly threefold more mutations were present among coevolved prey genomes than in control prey (Fig. 4). Predation may have increased the proportion of prey mutations that are beneficial, the average magnitude of fitness benefits conferred by adaptive mutations or the basal mutation rate of prey, although we consider the latter hypothesis unlikely in the absence of mutator mutations. Predation might also have increased total mutation supply if co-evolving prey underwent more sequential generations of growth per cycle than control prey due to predatory killing, but mutation supply is also determined by the size of transferred populations and control-evolved prey population sizes were threefold higher than coevolved prey populations on average (Supplementary Fig. 6).

The appearance of mutator clones exclusively in three coevolution communities (two in predator populations and one in prey) suggests that predatory interactions in such communities may increase the average benefit/cost ratio of mutator alleles^41^ relative to predation-free environments. Consistent with this hypothesis, mismatch-repair mutations (including one in *mutS*) also evolved in less that 200 generations in 25% of replicate populations of *Pseudomonas fluorescens* coevolving with a lytic phage, but not in phage-free control populations^42^. Mutator mutations may be at least transiently adaptive at the lineage level in some novel or highly variable environments by generating mutations beneficial at the individual level (including those contributing to ‘epistatic adaptations’ involving multiple mutations^43^) at a higher rate than non-mutators^41^, a scenario that may be promoted by predator–prey interactions.

Strong patterns of parallel genomic evolution provide compelling molecular evidence of adaptation specific to the predator–prey coevolution treatment. On the predator side, all coevolved populations gained mutations in a gene (*Mxan_RS27920*, hereby named *eatB* for ‘eat bacteria’) that is predicted to encode a membrane protein belonging to the major facilitator superfamily^44^. Intriguingly, homologs of *eatB* are detected only in species within the suborder Cystobacterineae, suggesting a recent evolutionary origin of this gene within the myxobacteria (Myxococcales order) (Supplementary Table 9). *eatB* is predicted to be transcribed within an operon composed of two genes, neither of which has previously been associated with predation or any other function. Future investigation of how mutations in *eatB* enhance fitness during consumption of *E. coli* may provide novel insights into the molecular mechanisms of *M. xanthus* predation.

Coevolved prey also displayed high genomic parallelism, with all but one population mutated at the outer-membrane protease gene *ompT*, a known virulence locus among uropathogenic *E. coli*^40^. The profile of mutations at this locus (Supplementary Table 5) indicates altered or lost gene function. Competition experiments with an *ompT* deletion mutant confirmed that loss of this gene conferred a fitness advantage specifically in the presence of predators (Fig. 3c). Given that OmpT deactivates some antimicrobial peptides produced by mammalian hosts^40^, it is unclear how its deactivation increases *E. coli* fitness during predation by *M. xanthus*. Intriguingly, antimicrobial activity by a zebrafish ribonuclease is activated by OmpT-mediated^45^ cleavage. It is thus conceivable that functional OmpT might activate a latent predation-enhancing function by cleaving an extracellular *M. xanthus* peptide.

The selection hotspot loci in predators (*eatB*) and prey (*ompT*) exhibit distinct frequency patterns among the three-clone sets we genome-sequenced from each population at the final evolutionary time-point. In 11/12 coevolved *M. xanthus* populations, all three clones carry an *eatB* mutation (Fig. 4b, Supplementary Table 6), which suggests that *eatB* mutants may have reached or approached fixation in most populations (although the possibility of ancestral alleles persisting at low frequencies cannot be excluded without further sampling). In turn, such fixation would suggest the operation of directional selection, which is characteristic of both “arms race”^5^ (aka “escalatory”) and “chase” forms of Red Queen dynamics (which differ in other respects)^13^. In contrast, among the sequenced three-clone sets from each of twelve coevolved *E. coli* populations, all but two are polymorphic at *ompT* (Fig. 4a, Supplementary Table 5). (Among the other two populations, one set had no *ompT* mutants and the other had three.) These *ompT* polymorphisms might reflect a snapshot of many populations simultaneously undergoing selective sweeps that were not yet complete. Alternatively, the prevalence of polymorphisms across populations suggests that ancestral and derived *ompT* alleles may be under fluctuating selection, which represents another form of Red Queen coevolution^11,13,46^. Multiple forms of Red Queen can operate in the same community over time^11^, but analysis of fitness patterns and genomic evolution at one evolutionary time point does not allow a clear test among them. However, the experimental system introduced here potentiates the future use of time-shift competition experiments^11^ and temporally extended analysis of genetic evolution to examine the character, dynamics and genetic basis of Red Queen predator–prey coevolution involving generalist bacterial predators.

Theory predicts and empirical studies of both macroorganisms in natural communities^47–49^ and microbes in experimental communities have confirmed that predation pressure can strongly influence (and often increases) prey diversity. Such effects have been observed among bacterial prey in response to protists^50^, the specialist bacterial predator *Bdellovibrio bacteriovorus*^27^ and phage^28^. In our experiments, mucoidy—a virulence factor in some human pathogens^38^—evolved preferentially and pervasively as a minority phenotype among prey exposed to *M. xanthus* predation, showing that a generalist bacterial predator drives sympatric phenotype diversification of its prey. Intriguingly, mucoid colony phenotypes have also evolved in response to interaction with lytic phages^39^, macrophages^38^ and antibiotics^51^ and have been associated with increased survival of protist grazing^52^ connecting this phenotypic state to an extremely divergent range of antagonistic interactions. Our finding that multiple prey traits associated with virulence in pathogens (OmpT and mucoidy) were targets of adaptation specifically among coevolved prey suggests that virulence-trait evolution may often be indirectly shaped by selective forces unrelated to pathogenesis^53^.

It will be of interest for future studies to systematically compare how individual bacterial prey species respond evolutionarily to distinct predators that differ greatly in their mechanisms of predation, life-history traits and metabolic niche. Some evolutionary responses such as mucoidy (or other alterations of the extracellular matrix) might jointly provide protection from multiple predator types due to a general effect of decreasing access of predatory weapons to the prey cell surface^54^. Other prey responses have also been found to synergistically carry-over across highly divergent predators. For example, in one study, predatory selection by protists indirectly decreased prey susceptibility to phage^55^. And yet, the radically different modes of predation exhibited by predatory microbes can be expected to often generate predator-specific prey adaptations. For example, bacteria evolve at specific surface proteins in response to phage infection^56^ that may be unlikely to also be targets of selection in response to protists (which consume whole prey cells^16^) or myxobacteria, which secrete predatory antibiotics and lytic enzymes^21,33^. In contrast, protists can impose selection on prey cell size^50,57^ that is unlikely to be similarly exerted by bacterial predators or phage.

Coevolution in two-species bacterial communities rapidly induced major changes in rates and patterns of molecular evolution in both predators and prey and at multiple prey traits (OmpT and mucoidy) implicated in interactions with radically divergent antagonistic partners. In light of these powerful effects of predation in simple communities over a short evolutionary period, generalist predatory bacteria can be expected to drive diversification and shape the evolutionary dynamics of complex interaction networks in natural microbial communities^50^. Components of such networks include fitness relationships and competition modes among diverse prey species, interactions between prey and other categories of predators (e.g., protists and nematodes) and interactions among predators. Finally, the rapid evolutionary responses reported here suggest that experimental studies of bacterial predator–prey coevolution may helpfully inform considerations of using predatory bacteria as potential biocontrol agents in medicine^58,59^ and agriculture^60,61^.

## Methods

### Ancestral strains

Three subclones each of streptomycin-sensitive and resistant (K43R mutation in *rpsL*) variants of *E. coli* MG1655 (provided by Dr. Balazs Bogos) were isolated by streaking frozen glycerol stocks onto LB agar plates. These sub-clones were grown to stationary phase in LB medium (37 °C, 200 rpm for 8–10 h) and used to initiate 12 replicate populations coevolving with *M. xanthus* (two from each subclone) and six replicate prey-only populations (one from each sub-clone). LB broth and LB broth with agar (Lennox) were purchased as powders from Sigma-Aldrich and prepared as per manufacturer’s instructions.

*M. xanthus* strain DK3470 was used as the ancestral predator^62^. This strain has a mutation (previously inferred to be in or near the *dsp*^62^/*dif* ^63^ gene region) that greatly reduces extracellular matrix production^62^, and thus allows cultures to be readily dispersed in liquid buffer even after growth on an agar surface. Three subclones of rifampicin-sensitive and resistant variants of DK3470 were isolated from colonies grown in CTT soft 0.5% agar (10 g/l casitone, 10 mM Tris pH 8.0, 8 mM MgSO_4_, 1 mM KPO_4_) at 32 °C after dilution plating from mid-exponential phase 8 mL liquid CTT cultures (32 °C, 300 rpm). These subclones were isolated, stored frozen and used to initiate predator-only control populations—six on M9 minimal medium supplemented with glucose (or “prey-growth agar”, details below, one population from each DK3470 subclone), six on M9-casitone medium (details below), and 12 populations coevolving with *E. coli* on M9 prey-growth agar (two from each subclone, Supplementary Table 1). Three of the casitone predator-only populations were discarded due to contamination during evolution. The remaining three were analyzed.

### Predator–prey coevolution arena

The experimental coevolution protocol is summarized in Fig. 1a. Coevolution was performed in 50 mL flasks containing 8 mL of prey-growth agar solid media (1× M9 salts, 2 mM MgSO_4_, 0.1 mM CaCl_2_, 0.2% glucose, 1.5% agar). We adjusted the densities of *E. coli* and *M. xanthus* populations by resuspending, respectively, ~10^5^ *E. coli* cells and ~10^9^ *M. xanthus* per 50 µL in TPM buffer (10 mM Tris pH 8.0, 8 mM MgSO_4_, 1 mM KPO_4_). To initiate the coevolution populations, we mixed 50 µL each of adjusted prey and predator cultures and spread the total volume on prey-growth agar with 7–9 sterile glass beads. For prey-only controls, 50 µL of adjusted *E. coli* culture was mixed with 50 µL of TPM buffer. We set two types of predator-only controls by spreading the adjusted *M. xanthus* cultures with 50 µL of TPM buffer onto both prey-growth agar and onto medium identical to prey-growth agar except that glucose was replaced with 0.5% casitone (a pancreatic digest of casein that *M. xanthus* can utilize as a carbon-substrate for growth). All flasks dried in a laminar-flow hood for about 30 min before incubation at 32 °C, 90% rH for ~84 h.

After ~84 h of incubation, the predator–prey communities were harvested by adding 5 mL TPM buffer and shaking for ~15 min at 300 rpm at 32 °C until the cultures were well suspended and dispersed. Evolving cultures were then reduced by a factor of 100 by mixing 1% (50 µL) of the resuspended volume with 50 µL of TPM buffer and then spreading onto fresh prey-growth agar as described above to initiate a new cycle. The evolution experiment ran for 25 such cycles, with frozen stocks made of evolving populations and communities after every second cycle starting at cycle 0 (in 20% glycerol, stored at −80 °C) and after the terminal cycle. The size of each predator and prey population was estimated every second cycle by dilution-plating onto LB hard agar (on which *M. xanthus* DK3470 does not grow) for prey and into CTT soft agar containing 10 µg/mL gentamicin (in which *E. coli* does not grow) for predators. The presence of both predator and prey populations at consistent end-of-cycle densities through the experiment (Supplementary Fig. 6) indicate that both predator and prey populations averaged at least ~6.64 generations (log_2_(100)) of replacement growth per cycle.

### Post-evolution fitness assays: competition experiments and FreqSeq analysis

To assess reciprocal adaptation, we performed fitness assays by competing coevolved populations vs. control-evolved or ancestral populations (Fig. 1b, c, Supplementary Figs. 3 and 4). *E. coli* and *M. xanthus* were isolated from coevolved populations through inoculation in LB and gentamicin-CTT as described above. All competition experiments were performed in three temporally separate replicates with whole-population samples of evolved prey and/or predator populations.

Prey populations were grown in LB as described above. Cultures were adjusted to an optical density (OD_600_) of ~1.0 (~10^8^ cells/mL) and 500 µL each of competitors with opposite streptomycin-resistance marker types were mixed. We diluted these mixes (1:100) and inoculated 50 µL together with 50 µL of either a predator culture (~5 × 10^9^ cells in TPM) or TPM buffer and spread onto prey-growth agar as described above. Predator cultures (ancestral, control-evolved and coevolved) were incubated in liquid gentamicin-CTT (10 µg/mL, 32 °C, 300 rpm) for ~60 h, upon which they were diluted and grown for further 8–10 h to obtain mid-exponential cultures (OD_600_ ~0.5–0.8), which were subsequently centrifuged and resuspended in TPM buffer after supernatant removal.

All twelve coevolved and all 6 control-evolved prey populations were competed against the ancestral prey (of the opposite marker state) in the presence of all three predator categories (Supplementary Fig. 7). Preliminary results (two replicates) indicated that both control and coevolved populations increased greatly in fitness relative to the ancestors, suggesting general adaptation to the abiotic conditions of the prey-growth agar selection regime. To better resolve potential adaptive responses by the coevolved prey specific to the presence of predators, we competed coevolved prey directly with control-evolved prey in the same flask. Each of the control-evolved prey populations of one marker type was competed independently against two of the coevolved prey populations of the opposite marker type. Thus, each of the twelve coevolved prey populations was included in one competition pair whereas each control-evolved prey population was included in two. Each of these direct competitions was subjected to four predator treatments: (i) the sympatrically coevolved predator, (ii) a control predator population that evolved on casitone medium in the absence of prey, (iii) the ancestral predator, (iv) no predator. In addition, competition flasks were incubated at 32 °C, 90% rH for 84 h and harvested using the aforementioned protocol. We centrifuged 2 mL of resuspended culture (14,000*g*, 5 min) and stored the pellets at −20 °C until lysate preparation. Pellets of the initial mixes were obtained and stored similarly.

Because all predator-only populations inoculated in prey-growth agar went extinct and predators growing on casitone are not perfect controls for the coevolution treatment due to the substitution of carbon source, we competed coevolved predators with their ancestors to test for predator adaptation. In preliminary experiments, we found that predator populations initially decline substantially when transferred directly from CTT to prey-growth agar prior to subsequently growing on prey. Thus, for competition experiments we first pre-conditioned predator populations separately for three 84-h cycles on prey-growth agar in the presence of the prey used in the subsequent assay. After three cycles of acclimatization, populations were harvested as described before and stored at −20 °C for lysate preparation. We prepared competition mixes and inoculated 100 µL of each mix onto prey-growth agar. The competition experiment was conducted for another three cycles. The remainder of the initial (*T*_0_) mixes was spun down and stored at −20 °C for lysate preparation.

Lysates of both prey and predator competition assays were prepared using the Triton X-100 protocol described by Goldenberger et al.^64^. The resulting supernatant was used for the Multiplex FreqSeq library preparation described below.

We modified the FreqSeq method^37^ to introduce a second barcode which allows multiplexing. Multiplex FreqSeq is described in Supplementary Fig. 1 and the validation method in Supplementary Fig. 2. The single-nucleotide polymorphisms responsible for antibiotic sensitivity/resistance to streptomycin (*rpsL*) and rifampicin (*rpoB*) were used to distinguish competitors with FreqSeq analysis for *E. coli* and *M. xanthus*, respectively. A combination of two barcodes was used to label all populations involved in the competitions: each pair of right-side and left-side barcodes therefore identified one replicate of a given competition treatment. The primers and PCR conditions for Multiplex FreqSeq are detailed in Supplementary Tables 2 and 3. DNA libraries were normalized, pooled, and purified (Ampure XP beads) and their final sizes and concentrations were confirmed using an Agilent Bioanalyser. The pooled library was then diluted to 4 nM in water and run on a MiSeq machine at the Genomic Diversity Centre at ETH Zürich after mixing with 30% standard PhiX library. We ran one and nine MiSeq sequencing for prey and predator competitions, respectively. Data from MiSeq were checked for quality using FastQC and Illumina adapters were removed using Trimmomatic v0.33^65^ with the parameters: ILLUMINACLIP:adapter.fa:2:30:10, to leave the six nucleotide barcode at the 3′ end. The reads were demultiplexed using the barcode splitter algorithm in the FASTX-Toolkit (http://hannonlab.cshl.edu/fastx_toolkit/). Demultiplexed reads were stored as separate fastq files for each right-side barcode and analyzed using the Freq-Out program^37^ to obtain frequencies of each competitor from pre- and post-competition mixes. A bash script for running each step of data analysis in a single script is provided in the Supplementary Note 1. Frequency estimates from unexpected barcodes, with less than 10,000 reads or beyond the range tested in the validation assay (0.05–0.95, Supplementary Fig. 2) were discarded.

We calculated the relative fitness of the coevolved prey or predators using the formula of Ross-Gillespie et al.^66^

1 $$v = \frac{{x2\left( {1 - x1} \right)}}{{x1\left( {1 - x2} \right)}}$$

where *x*1 and *x*2 are initial and final frequencies of the coevolved cells, respectively.

### Phenotypic evolution of prey: mucoidy and predation resistance

To screen for mucoid variants at cycle 18, aliquots of frozen stocks of coevolved and control-evolved prey populations were thawed in 100 µL TPM buffer, dilution plated onto LB agar and examined for the presence of mucoid colonies after overnight incubation at 32 °C, 90% rH. We subsequently conducted a series of experiments to assess whether mucoidy may confer resistance against the predators.

First, we quantitatively assayed the efficacy of ancestral *M. xanthus* in reducing prey population size using one isolated mucoid clone and one non-mucoid clone from population ME4 (cycle 18). We compared the effect of *M. xanthus* on the end-of-cycle population sizes of different prey strains under the same conditions as in the evolution treatments that included prey (Fig. 2a). After 84 h, we assessed prey population size in the presence and absence of *M. xanthus* by dilution-plating on LB agar and calculated the percentage reduction of each type caused by the presence of predator. In experiments under the same conditions, we estimated exponential population growth rates of the same mucoid and non-mucoid strains in the absence of predator (from 5 to 20 h of growth), but no difference between the strains was detected (*r* *=* 0.52 and 0.5, respectively, two-sided *t* test: *t*_4_ = −0.2804, *p* *=* 0.79). In these experiments, multiple growth cultures were established for each strain within each replicate and population size at each time point was assessed by whole-culture destructive sampling.

We also measured the swarming rate of the predator on the different prey types (coevolved mucoid and non-mucoid isolates from ME4 and ancestral, Fig. 2b). We spotted ancestral *M. xanthus* (10 µL at 5 × 10^9^ cells/mL) on top of a 48-h old *E. coli* lawn on prey-growth agar and allowed it to swarm for 7 days (32 °C, 90% rH). Swarm edges were outlined after 1 day and 7 days, and migration distances were measured on opposite sides of at least three separate transects (thus at least six measurements per plate). For all assays, we performed three biological replicates with two technical replicates each.

Finally, we qualitatively assessed the ability of an expanding predator swarm to penetrate and lyse adjacent colonies of coevolved mucoid, coevolved non-mucoid and ancestral (non-mucoid) prey (Fig. 2c) for seven coevolved communities. For each, one coevolved mucoid clone, one coevolved non-mucoid clone and ancestral *E. coli* were grown in LB to OD_600_ ~1.0 and 10 µL were spotted on both prey-growth and on CTT agar at a 1-cm distance from a 10 µL spot of ancestral *M. xanthus* (5 × 10^9^ cells/mL). The plates were incubated (32 °C, 90% rH) and predator–prey colony interface phenotypes were observed every 24 h. The image shown in Fig. 2c was taken after 5 days of incubation.

### Whole-genome sequencing

After cycle 25 of the evolution experiment, three independent predator and/or prey clones each were randomly picked from either LB agar (prey), or gentamycin-CTT agar (predators) for all control-evolved and coevolved populations. Isolated predator and prey clones were grown to high density in 8 mL CTT and LB liquid medium, respectively, and centrifuged at 4000*g* for 15 min. Pellets were stored at −80°C until DNA was extracted. Whole-genomic DNA was isolated with Qiagen’s Genomic DNA extraction buffer kit and 20/G Genomic-tips. DNA quantity was checked with a Qubit fluorometer (Thermo Fisher Scientific). Genomes of predator and prey clones were sequenced on different Illumina® HiSeq® 2500 sequencing machines in paired-end mode; prey populations were processed by Fasteris (Geneva, Switzerland) producing reads of 125 bp while predator genomes were handled by the D-BSSE Quantitative Genomics Facility of ETH Zürich (Basel, Switzerland), also with read lengths of 125 bp.

Read quality was assessed using FastQC (http://www.bioinformatics.babraham.ac.uk/projects/fastqc/). Illumina-specific adapters/primers and low quality base calls were trimmed from reads using Trimmomatic v0.33^65^ with the following basic parameters: ILLUMINACLIP:Nextera + TruSeq3-PE2.fa:2:25:10 CROP:124 HEADCROP:5 LEADING:30 TRAILING:28 SLIDINGWINDOW:4:28 MINLEN:77. The trimmed reads from *E. coli* prey populations were subsequently mapped to the *E. coli* str. K-12 substr. MG1655 reference genome^67^ using breseq 0.27.0^68^. The latter step also involved error correction and variant calling (based on samtools v 1.3.1^69^; see Supplementary Tables 5 and 7 for a mutation summary for all prey populations). The trimmed reads for all the predator clones were mapped to a modified version of the reference genome of *M. xanthus* str. DK1622 which contains all mutations present in the experiment’s founding clone, DK3470, relative to its parent strain, *M. xanthus* str. DK1622 (**refseq**: NC_008095). The operonic context of *eatB* was predicted at http://www.microbesonline.org/operons/gnc246197.html.

### Inference of the reference genome for *M. xanthus* DK3470

To infer the reference genome of the ancestral DK3470 clone, we first relied on a whole genome assembly of the same Illumina® reads using SPAdes v. 3.11.1 (with parameters -k 21,33,55,77 --careful), from which we detected the previously reported Tn5-transposon that inserted in the coding region of *Mxan_RS3243549* and includes the transposase and genes conferring resistance to the antibiotics neomycin/kanamycin, bleomycin and streptomycin^70^. That transposon was previously reported to have co-localized with an unknown additional mutation, responsible for the non-cohesive growth of DK3470^62^. We identified this unknown mutation by mapping the trimmed Illumina® reads against the published reference genome of isogenic *M. xanthus* str. DK1622 using breseq 0.27.0. We find that the mutation is a single base-pair deletion in an intergenic region, 43 bp downstream of Mxan_RS32420 (*difA*) and 1386 bp upstream of Mxan_RS32425 (*thiL*). This mutation is also present in all evolved *M. xanthus* descendants sequenced for this study. We included these two positional differences in the reference *M. xanthus* strain DK1622 and used that modified sequence for mapping of our predators.

### *ΔompT* mutant construction

Δ*ompT-*mutants were constructed by replacing the coding sequence of the *ompT* gene with a kanamycin resistance marker via recombineering^71^. The streptomycin-sensitive and resistant ancestral *E. coli* strains were transformed with the recombineering plasmid pSIM6 and maintained at 30°C degrees with 100 mg/L ampicillin in the media. The kanamycin marker was amplified from the KEIO strain JW0554^72^ with PCR primers *ompT*-F (GTTACATTGAAATGGCTAGTTATTCCCC) and *ompT*-R (CAGTGGAGCAATATGTAATTGACTC). The purified PCR product was then used to replace *ompT* in both *E. coli* strains with pSIM6 (for a detailed recombineering protocol, see^71^). Positive clones were confirmed by growth on media with 100 mg/L kanamycin and sequencing with the aforementioned primers. To ensure a clean genetic background, P1 transduction was performed using the constructed *ΔompT* strains as donor and the ancestral strains as recipient^73^. Positive-P1 transductants were confirmed by kanamycin resistance, colony-PCR and sequencing. All the subsequent fitness assays were performed using the P1-transduced strains.

### Relative fitness of *ΔompT* prey

We estimated the relative fitness of Δ*ompT* prey in competition with ancestral *E. coli* under the conditions of the post-evolution fitness assays (Fig. 3c, Supplementary Fig. 5). Wildtype strains competed against Δ*ompT* mutant of opposite marker for one cycle (84 h) in the prey-growth medium in the presence and in the absence of ancestral predators. Initial and final frequencies of each strain were determined by dilution-plating onto LB agar (plain and supplemented with 100 µg/mL streptomycin). To control for any marker effect, we also conducted competitions with clones of the opposite marker type but same *ompT* genotype. Relative fitness was calculated as described above. At least six independent replicates for each competition were performed.

### Statistical analyses

We analyzed relative fitness of prey and predators with analysis of variance (ANOVA type II). For coevolved prey, we used predator treatment (coevolved, control-evolved, ancestral or no predator), population ID (ME1 to ME12) and competitor ID (control-evolved *E. coli* E1–E6) as factors. For coevolved predators, we used prey treatment (coevolved, ancestral or casitone), population ID (ME1–ME12) and competitor ID (rifampicin-resistant or sensitive ancestor) as factors. Post hoc tests were Tukey-tests for multiple comparisons and one-sample *t* tests with Holm–Bonferroni corrections. For the *t* tests, data were the mean of three replicate competitions per coevolved population so that the unit of replication was each coevolved population (*n* = 12). For the prey *ompT* mutant, explanatory factors were predator treatment (presence or absence), time of the experiment (day 1 or day 2) and the resistance phenotype of the mutant (streptomycin resistant or sensitive). We log-transformed all relative fitness data to meet the assumption of normality. We tested for differences in the rate of molecular evolution between coevolved vs control-evolved populations using one-sided, two-sample *t* tests. Evolutionary rate was quantified as the average number of mutations found across all populations within each treatment (with each population represented by two or three independently isolated and sequenced clones) after 25 cycles of evolution. Statistical analyses were performed using R studio (version 1.0.136)^74^ and the packages car^75^ and ggplot^76^.

### Reporting summary

Further information on research design is available in the Nature Research Reporting Summary linked to this article.

## Supplementary information

Supplementary Information

Reporting Summary

Source Data

## Data Availability

Genome sequences have been deposited in the SRA database under BioProject accession PRJNA551936 (BioSample accessions SAMN12169214–SAMN12169315). All other data generated or analyzed during this study are included in this published article (and its Supplementary information files). The source data underlying Figs. 1b, c, 2a, b, 3c, 4, and Supplementary Figs. 1–3 and 5–7 are provided as a Source Data file.

## References

[1] Leibold MA, Chase JM, Shurin JB, Downing AL (1997). Species turnover and the regulation of trophic structure. Annu. Rev. Ecol. Syst..

[2] Hairston NG, Smith FE, Slobodkin LB (1960). Community structure, population control, and competition. Am. Nat..

[3] Estes James, Crooks Kevin, Holt Robert D. (2013). Predators, Ecological Role of. Encyclopedia of Biodiversity.

[4] Sinclair A. R. E., Mduma Simon, Brashares Justin S. (2003). Patterns of predation in a diverse predator–prey system. Nature.

[5] Brodie III ED, Brodie ED, Brodie ED (1999). Predator-prey arms races. Bioscience.

[6] Hass CC, Valenzuela D (2002). Anti-predator benefits of group living in white-nosed coatis (*Nasua narica*). Behav. Ecol. Sociobiol..

[7] Stevens M, Merilaita S (2009). Animal camouflage: current issues and new perspectives. Philos. Trans. R. Soc. B Biol. Sci..

[8] Matz C (2008). Marine biofilm bacteria evade eukaryotic predation by targeted chemical defense. PLoS One.

[9] Estes James A., Burdin Alexander, Doak Daniel F. (2015). Sea otters, kelp forests, and the extinction of Steller’s sea cow. Proceedings of the National Academy of Sciences.

[10] Van Valen L (1973). A new evolutionary law. Evol. Theory.

[11] Hall AR, Scanlan PD, Morgan AD, Buckling A (2011). Host-parasite coevolutionary arms races give way to fluctuating selection. Ecol. Lett..

[12] Brockhurst MA, Koskella B (2013). Experimental coevolution of species interactions. Trends Ecol. Evol..

[13] Brockhurst MA (2014). Running with the Red Queen: the role of biotic conflicts in evolution. Proc. R. Soc. B Biol. Sci..

[14] Van Valen L (1974). Molecular evolution as predicted by natural selection. J. Mol. Evol..

[15] Paterson S (2010). Antagonistic coevolution accelerates molecular evolution. Nature.

[16] Fenchel Tom (1987). Ecology of Protozoa.

[17] Simek K, Chrzanowski TH (1992). Direct and indirect evidence of size-selective grazing on pelagic bacteria by freshwater nanoflagellates. Appl. Environ. Microbiol..

[18] Pérez J, Moraleda-Muñoz A, Marcos-Torres FJ, Muñoz-Dorado J (2016). Bacterial predation: 75 years and counting!. Environ. Microbiol..

[19] Jurkevitch, E. *Predatory prokaryotes: biology, ecology and evolution*. (Springer Science & Business Media, 2006).

[20] Reichenbach H (1999). The ecology of the myxobacteria. Environ. Microbiol..

[21] Muñoz-Dorado, J., Marcos-Torres, F. J., García-Bravo, E., Moraleda-Muñoz, A. & Pérez, J. Myxobacteria: moving, killing, feeding, and surviving together. *Front. Microbiol*. **7**, 781 (2016). 10.3389/fmicb.2016.00781 PMC4880591 27303375

[22] Morgan AD, MacLean RC, Hillesland KL, Velicer GJ (2010). Comparative analysis of myxococcus predation on soil bacteria. Appl. Environ. Microbiol..

[23] Mendes-Soares H, Velicer GJ (2013). Decomposing predation: testing for parameters that correlate with predatory performance by a social bacterium. Microb. Ecol..

[24] Jurkevitch, E. A brief history of short bacteria: a chronicle of *Bdellovibrio* (and like organisms) research in *Predatory Prokaryotes*. (eds Jurkevitch, E. & Steinbüchel A.) **4**, 1–9 (Springer, Berlin, Heidelberg, 2007).

[25] de Jonge Patrick A., Nobrega Franklin L., Brouns Stan J.J., Dutilh Bas E. (2019). Molecular and Evolutionary Determinants of Bacteriophage Host Range. Trends in Microbiology.

[26] Huang, W., Traulsen, A., Werner, B., Hiltunen, T. & Becks, L. Dynamical trade-offs arise from antagonistic coevolution and decrease intraspecific diversity. *Nat. Commun*. 10.1038/s41467-017-01957-8 (2017). 10.1038/s41467-017-01957-8 PMC5727225 29233970

[27] Gallet R (2007). Predation and disturbance interact to shape prey species diversity. Am. Nat..

[28] Brockhurst MA, Morgan AD, Fenton A, Buckling A (2007). Experimental coevolution with bacteria and phage. Infect. Genet. Evol..

[29] Hillesland KL, Velicer GJ, Lenski RE (2009). Experimental evolution of a microbial predator's ability to find prey. Proc. Biol. Sci..

[30] Kaper JB, Nataro JP, Mobley HLT (2004). Pathogenic *Escherichia coli*. Nat. Rev. Microbiol..

[31] Ishii S, Ksoll WB, Hicks RE, Sadowsky MJ (2006). Presence and growth of naturalized *Escherichia coli* in temperate soils from Lake Superior watersheds. Appl. Environ. Microbiol..

[32] Marshall RC, Whitworth DE (2019). Is “Wolf‐Pack” predation by antimicrobial bacteria cooperative? Cell behaviour and predatory mechanisms indicate profound selfishness, even when working alongside kin. BioEssays.

[33] Berleman JE (2014). The lethal cargo of *Myxococcus xanthus* outer membrane vesicles. Front. Microbiol..

[34] Shimkets LJ (1999). Intercellular signaling during fruiting-body development of *Myxococcus xanthus*. Annu. Rev. Microbiol..

[35] Rosenberg E, Keller KH, Dworkin M (1977). Cell density dependent growth of *Myxococcus xanthus* on casein. J. Bacteriol..

[36] Velicer GJ, Vos M (2009). Sociobiology of the Myxobacteria. Annu. Rev. Microbiol..

[37] Chubiz LM, Lee M-C, Delaney NF, Marx CJ (2012). FREQ-Seq: a rapid, cost-effective, sequencing-based method to determine allele frequencies directly from mixed populations. PLoS One.

[38] Miskinyte M (2013). The genetic basis of *Escherichia coli* pathoadaptation to macrophages. PLoS Pathog..

[39] Scanlan PD, Buckling A (2012). Co-evolution with lytic phage selects for the mucoid phenotype of *Pseudomonas fluorescens* SBW25. ISME J..

[40] Hui C-Y (2010). *Escherichia coli* outer membrane protease OmpT confers resistance to urinary cationic peptides. Microbiol. Immunol..

[41] Giraud A (2001). Costs and benefits of high mutation rates: adaptive evolution of bacteria in the mouse gut. Science.

[42] Pal C, Maciá MD, Oliver A, Schachar I, Buckling A (2007). Coevolution with viruses drives the evolution of bacterial mutation rates. Nature.

[43] Wistrand-Yuen E (2018). Evolution of high-level resistance during low-level antibiotic exposure. Nat. Commun..

[44] Reddy Vamsee S., Shlykov Maksim A., Castillo Rostislav, Sun Eric I., Saier Milton H. (2012). The major facilitator superfamily (MFS) revisited. FEBS Journal.

[45] Zanfardino A, Pizzo E, Di Maro A, Varcamonti M, D’Alessio G (2010). The bactericidal action on Escherichia coli of ZF-RNase-3 is triggered by the suicidal action of the bacterium OmpT protease. FEBS J..

[46] Stenseth NC, Smith JM (1984). Coevolution in ecosystems: red queen evolution or stasis?. Evolution.

[47] Paine RT (1969). A note on trophic complexity and community stability. Am. Nat..

[48] Nosil P., Crespi B. J. (2006). Experimental evidence that predation promotes divergence in adaptive radiation. Proceedings of the National Academy of Sciences.

[49] Vamosi Steven M (2005). On the role of enemies in divergence and diversification of prey: a review and synthesis. Canadian Journal of Zoology.

[50] Johnke J (2017). A generalist protist predator enables coexistence in multitrophic predator-prey systems containing a phage and the bacterial predator bdellovibrio. Front. Ecol. Evol..

[51] Piña SE, Mattingly SJ (1997). The role of fluoroquinolones in the promotion of alginate synthesis and antibiotic resistance in *Pseudomonas aeruginosa*. Curr. Microbiol..

[52] Matz C, Deines P, Jürgens K (2002). Phenotypic variation in Pseudomonas sp. CM10 determines microcolony formation and survival under protozoan grazing. FEMS Microbiol. Ecol..

[53] Sun S, Noorian P, McDougald D (2018). Dual role of mechanisms involved in resistance to predation by protozoa and virulence to humans. Front. Microbiol..

[54] Scholl D, Adhya S, Merril C (2005). *Escherichia coli* K1’s capsule is a barrier to bacteriophage T7. Appl. Environ. Microbiol..

[55] Örmälä-Odegrip A-M (2015). Protist predation can select for bacteria with lowered susceptibility to infection by lytic phages. BMC Evol. Biol..

[56] Seed Kimberley D. (2015). Battling Phages: How Bacteria Defend against Viral Attack. PLOS Pathogens.

[57] Sherr EB, Sherr BF (2002). Significance of predation by protists in aquatic microbial food webs. Antonie Van Leeuwenhoek.

[58] Willis AR (2016). Injections of predatory bacteria work alongside host immune cells to treat shigella infection in Zebrafish Larvae. Curr. Biol..

[59] Kadouri DE, To K, Shanks RMQ, Doi Y, Shutt KA (2013). Predatory bacteria: a potential ally against multidrug-resistant gram-negative pathogens. PLoS One.

[60] Scherff RH (1973). Control of bacterial blight of soybean by Bdellovibrio bacteriovorus. Phytopathology.

[61] Bull CT, Shetty KG, Subbarao KV (2002). Interactions between myxobacteria, plant pathogenic fungi, and biocontrol agents. Plant Dis..

[62] Shimkets LJ (1986). Role of cell cohesion in *Myxococcus xanthus* fruiting body formation. J. Bacteriol..

[63] Yang Z., Ma X., Tong L., Kaplan H. B., Shimkets L. J., Shi W. (2000). Myxococcus xanthus dif Genes Are Required for Biogenesis of Cell Surface Fibrils Essential for Social Gliding Motility. Journal of Bacteriology.

[64] Goldenberger D, Perschil I, Ritzler M, Altwegg M (1995). A simple ‘universal’ DNA extraction procedure using SDS and proteinase K is compatible with direct PCR amplification. PCR Methods Appl..

[65] Bolger AM, Lohse M, Usadel B (2014). Trimmomatic: a flexible trimmer for Illumina sequence data. Bioinformatics.

[66] Ross-Gillespie A, Gardner A, West SA, Griffin AS (2007). Frequency dependence and cooperation: theory and a test with bacteria. Am. Nat..

[67] Blattner FR (1997). The complete genome sequence of *Escherichia coli* K-12. Science.

[68] Barrick JE (2014). Identifying structural variation in haploid microbial genomes from short-read resequencing data using breseq. BMC Genom..

[69] Li H. (2011). A statistical framework for SNP calling, mutation discovery, association mapping and population genetical parameter estimation from sequencing data. Bioinformatics.

[70] Kuspa A (1989). Genes required for developmental signalling in *Myxococcus xanthus*: three asg loci. J. Bacteriol..

[71] Sharan Shyam K, Thomason Lynn C, Kuznetsov Sergey G, Court Donald L (2009). Recombineering: a homologous recombination-based method of genetic engineering. Nature Protocols.

[72] Baba T (2006). Construction of *Escherichia coli* K-12 in-frame, single-gene knockout mutants: the Keio collection. Mol. Syst. Biol..

[73] Thomason, L. C., Costantino, N. & Court, D. L. *E. coli* genome manipulation by P1 transduction. in *Current Protocols in Molecular Biology* **Chapter 1**, 1.17.1–1.17.8 (John Wiley & Sons, Inc., 2007). 10.1002/0471142727.mb0117s79 18265391

[74] Dayal Vikram (2015). An Introduction to R for Quantitative Economics.

[75] Fox, J. et al. An R Companion to Applied Regression, Second Edition. *R topics documented* (2014).

[76] Bodenhofer U., Kothmeier A., Hochreiter S. (2011). APCluster: an R package for affinity propagation clustering. Bioinformatics.

