# Supplementary material for: Bacterial predator-prey coevolution accelerates genome evolution and selects on virulence-associated prey defences
Source: Nat Commun. 2019 Sep 20;10:4301. doi: 10.1038/s41467-019-12140-6 (PMC6754418; doi:10.1038/s41467-019-12140-6)
Supplement: Supplementary file 1 — Supplementary Information [file 41467_2019_12140_MOESM1_ESM.pdf]

Bacterial predator-prey coevolution accelerates genome evolution and selects on virulence-associated prey defences

Supplementary Information

Nair, Vasse *et al*.

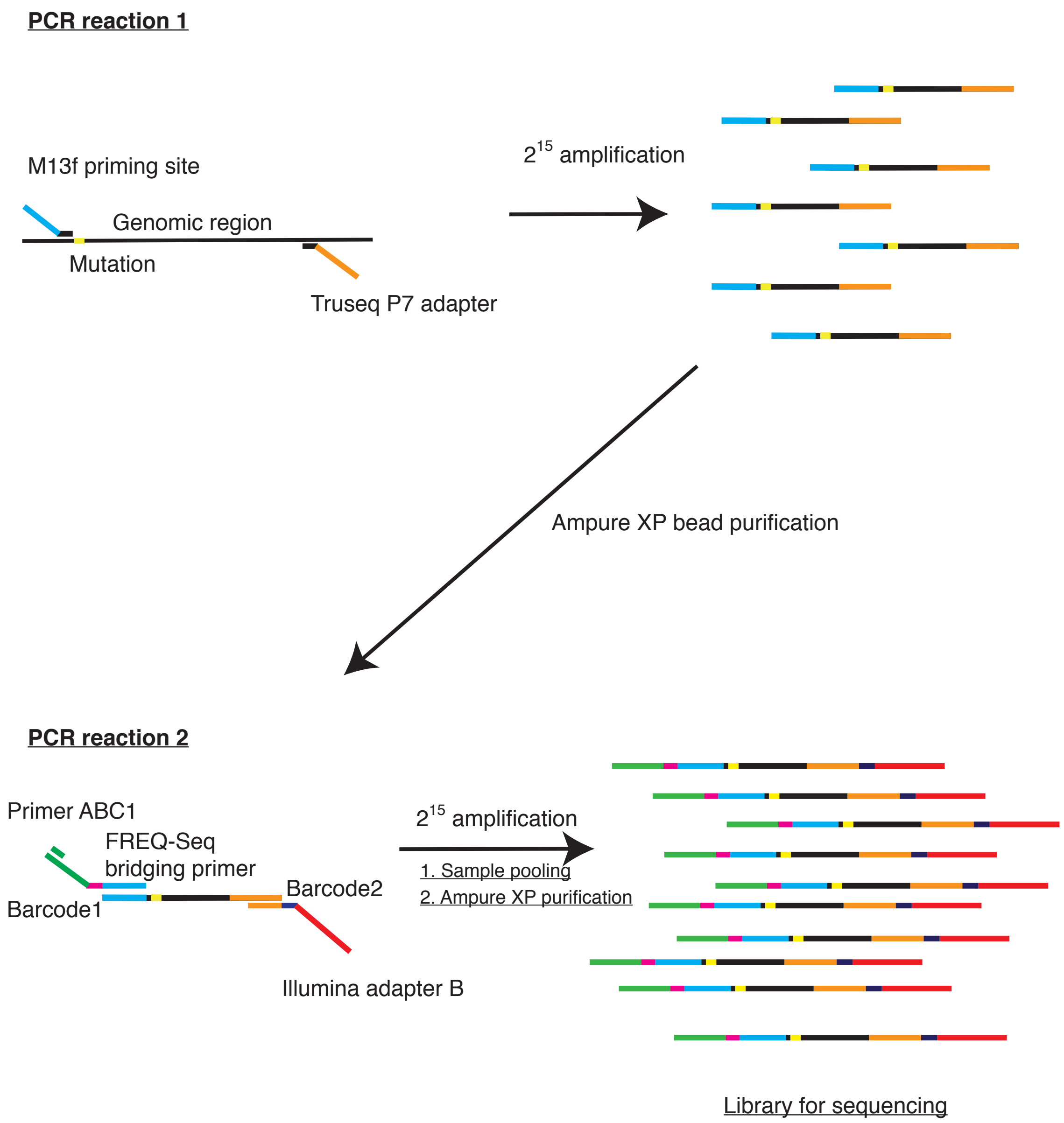

Supplementary Figure 1. Schematic representation of Multiplex FreqSeq. Adapted from Chubiz *et al* (2012)[1] by RRN and GJV. The bridging primer is made as in original reference. The first PCR reaction amplifies the genomic region that contains the mutation (yellow) identifying the two competitors. The reaction adds a M13f tail (blue) to the 5' end and a P7 tail (orange) to the 3' end that act as primers of next PCR reaction. The PCR products are purified using Ampure XP beads. The second PCR reaction adds that two barcodes (pink and dark blue) to the amplicon in a reaction involving three primers. The primer ABC1 (green) amplifies the bridging primer (blue) which in turn binds to the M13f priming site of the template DNA. The bridging primer along with the reverse primer (orange) adds the two barcodes to the amplicon. The samples are then pooled and purified to be used for Illumina sequencing.

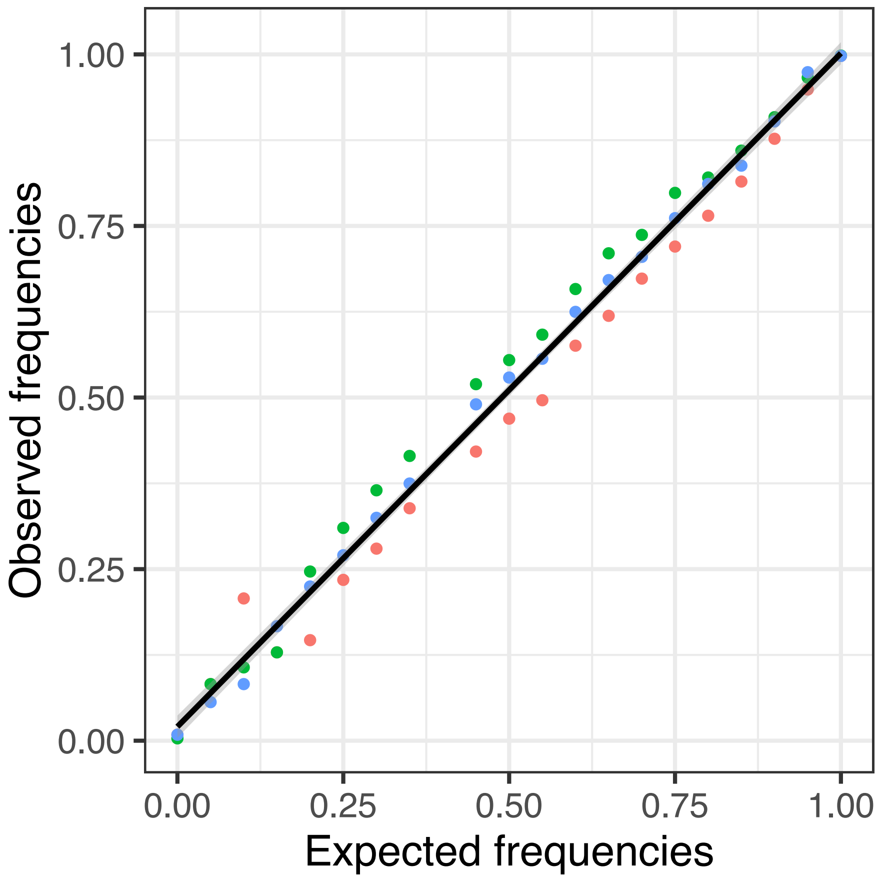

Supplementary Figure 2. Validation of Multiplex Freqseq. Streptomycin-resistant and sensitive strains of *E. coli* MG1655 were grown overnight, adjusted to an OD of 1.0 and mixed in different ratios before being spun down and lysed to obtain DNA for frequency analysis by Multiplex Freqseq. The original tubes used to make the mixes were dilution plated to confirm they had equal densities. The grey ribbon around the line depicts the 95% confidence region of the linear fit to the data and each color corresponds to a replicate (n = 3). Source data are provided as a Source Data file.

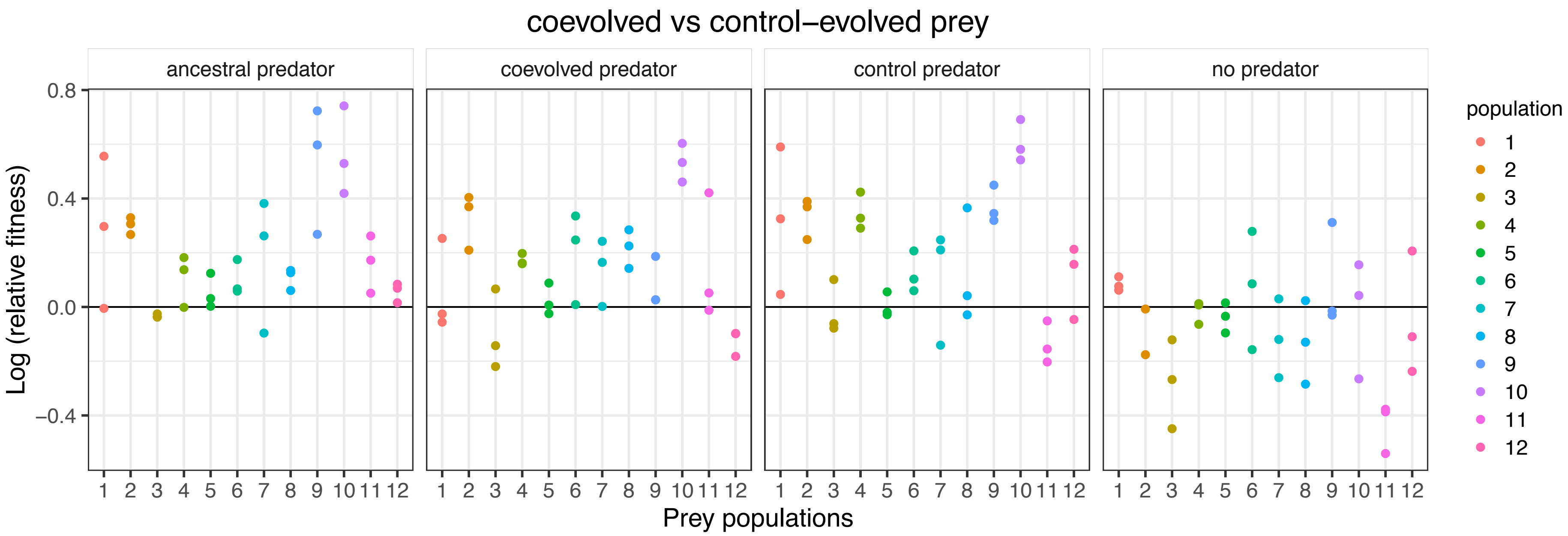

Supplementary Figure 3. Prey adaptation to predation. Fitness of each coevolved prey population relative to control-evolved prey in direct competition experiments in the presence and absence of predators. Each colour corresponds to one coevolved prey population and each dot is a replicate (n = 3 for most populations). Source data are provided as a Source Data file.

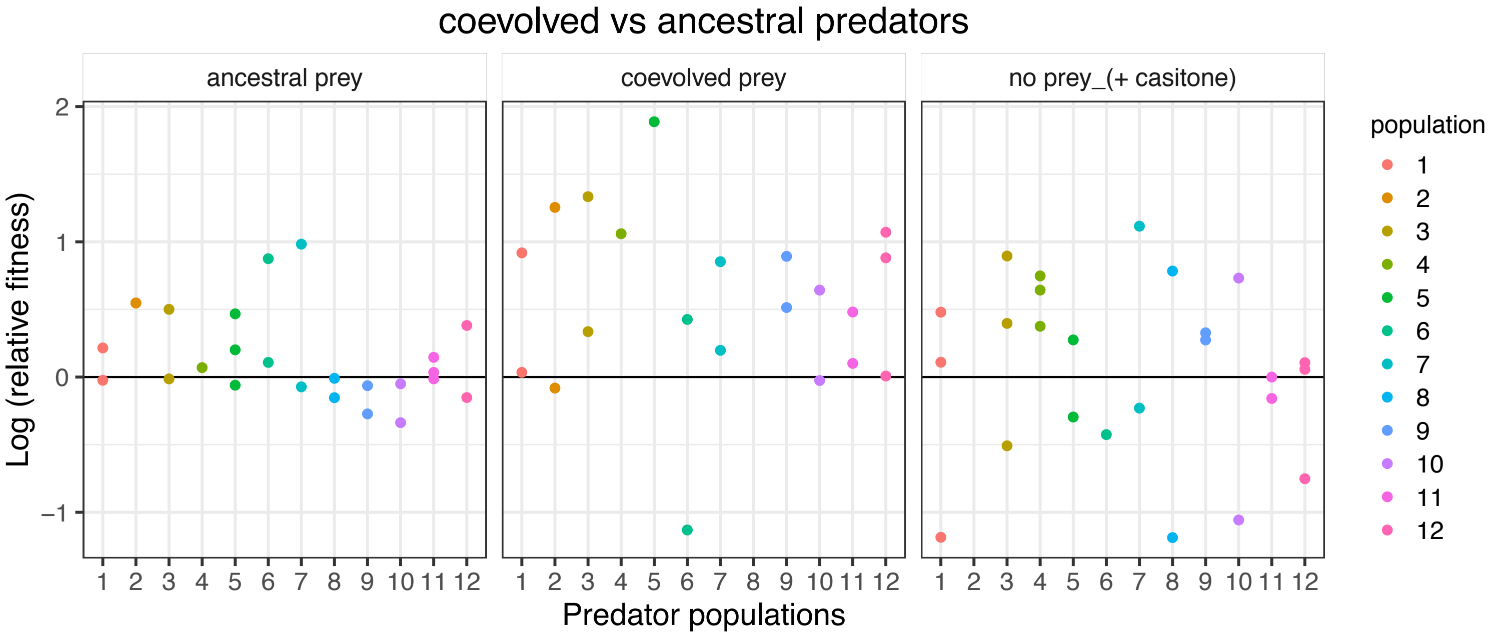

Supplementary Figure 4. Predator adaptation in the coevolution treatment. Fitness of each coevolved predator population relative to its reciprocally marked ancestor in direct competitions in the presence of prey and on casitone in the absence of prey. Each colour corresponds to one coevolved predator population and each dot is a replicate (n = 3 for most populations). Source data are provided as a Source Data file.

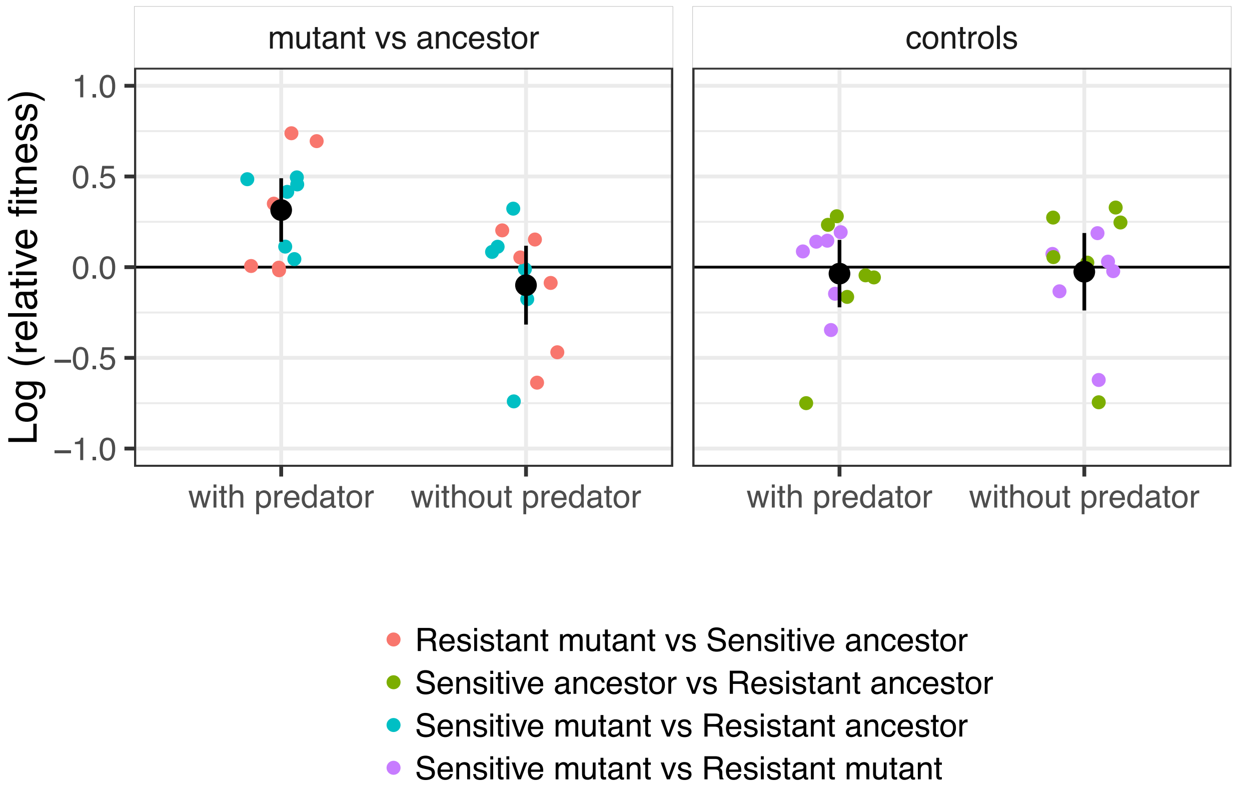

Supplementary Figure 5. Fitness effect of *ompT* deletion. Fitness of an *ompT* deletion prey mutant in competition with the ancestral prey in the presence and absence of the ancestral predator (n = 6 for both treatments). Results of control competitions are also shown. Black dots are treatment means and error bars show 95% confidence intervals (*t* distribution). Source data are provided as a Source Data file.

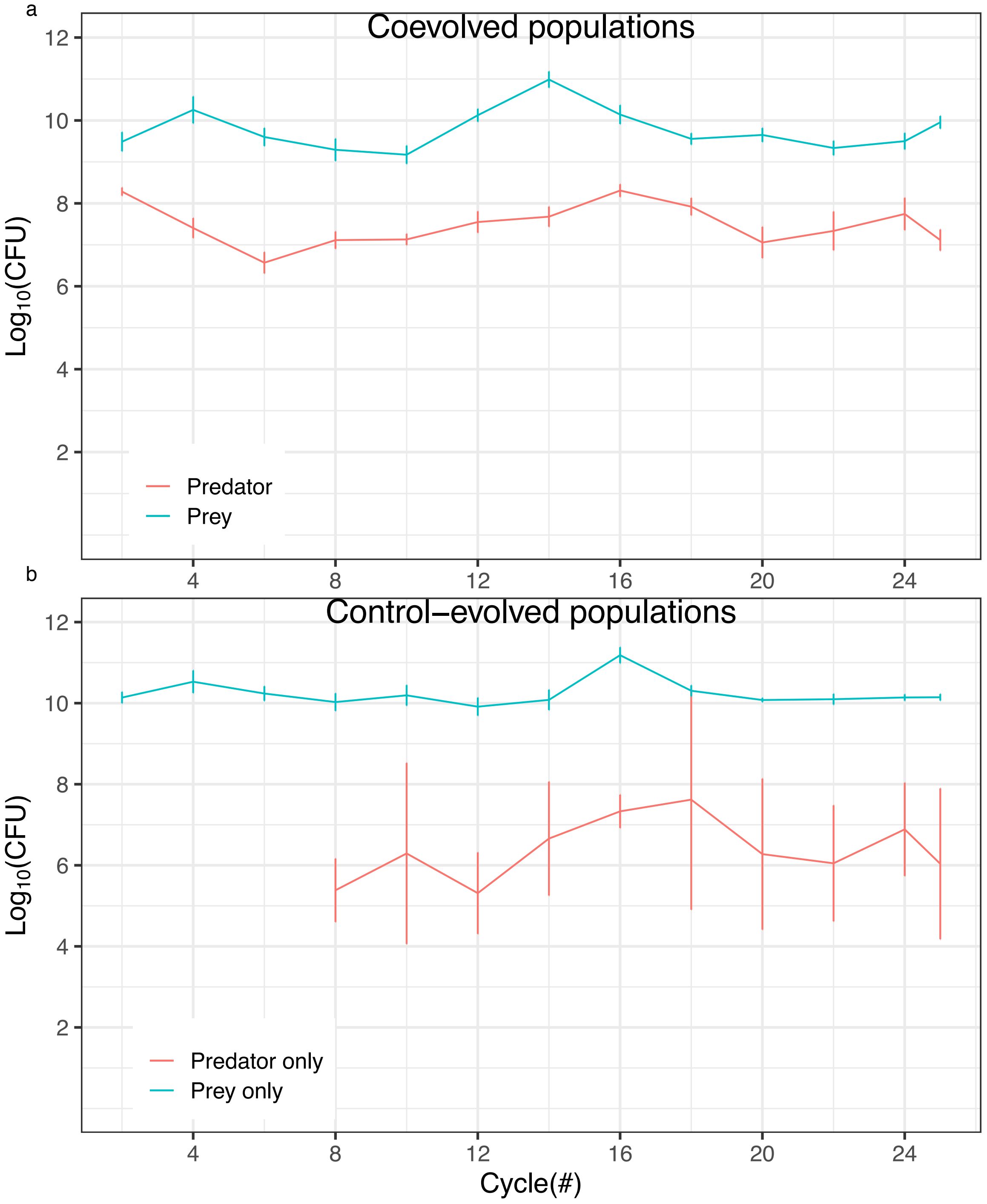

Supplementary Figure 6. End-of-cycle population sizes. Average size of predator (red) and prey (green) populations across all replicate (a) coevolution populations (n = 12) and (b) control-evolved populations (n = 6 for control-evolved prey and n = 3 for control-evolved predators grown on casitone medium). Error bars depict 95% confidence intervals (*t* distribution). Source data are provided as a Source Data file.

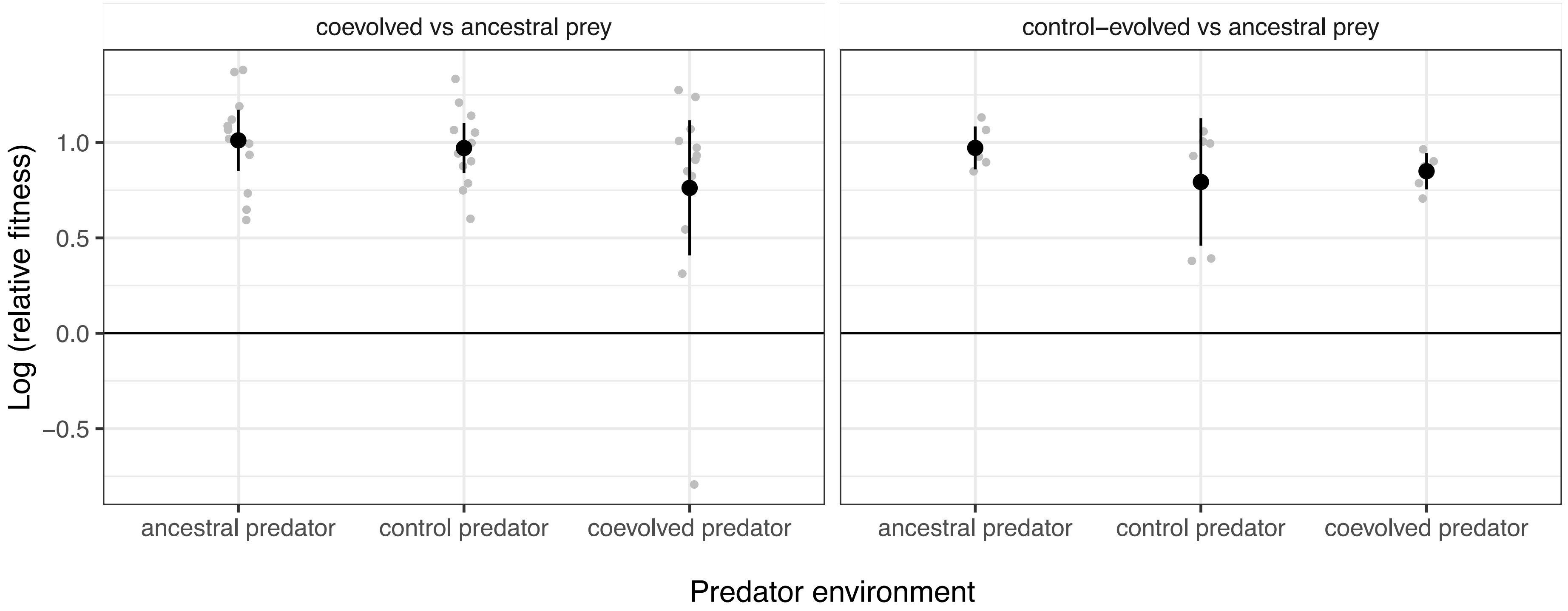

Supplementary Figure 7. Fitness of evolved prey relative to ancestors. Fitness of each coevolved and control-evolved prey population relative to their reciprocally marked ancestors in the presence of three different predator types. Grey dots are population means, black dots are treatment means and error bars show 95% confidence intervals (n = 2, *t* distribution). Source data are provided as a Source Data file.

Supplementary Table 1. Composition of the populations in the evolution experiment. Strep + (-) corresponds to streptomycin-resistant (sensitive) *E. coli* and Rif + (-) to rifampicin-resistant (sensitive) *M. xanthus*.

Population ID	Treatment	Composition	*E. coli*	*M. xanthus*
ME1	coevolved	ES1 – MS1	Strep -	Rif -
ME2	coevolved	ER1 – MR1	Strep +	Rif +
ME3	coevolved	ES2 – MS2	Strep -	Rif -
ME4	coevolved	ER2 – MR2	Strep +	Rif +
ME5	coevolved	ES3 – MS3	Strep -	Rif -
ME6	coevolved	ER3 – MR3	Strep +	Rif +
ME7	coevolved	ES1 – MR1	Strep -	Rif +
ME8	coevolved	ER1 – MS1	Strep +	Rif -
ME9	coevolved	ES2 – MR2	Strep -	Rif +
ME10	coevolved	ER2 – MS2	Strep +	Rif -
ME11	coevolved	ES3 – MR3	Strep -	Rif +
ME12	coevolved	ER3 – MS3	Strep +	Rif -
E1	control-evolved	ES1	Strep -	-
E2	control-evolved	ER1	Strep +	-
E3	control-evolved	ES2	Strep -	-
E4	control-evolved	ER2	Strep +	-
E5	control-evolved	ES3	Strep -	-
E6	control-evolved	ER3	Strep +	-
M1	control-evolved	MS1	-	Rif -
M2	control-evolved	MR1	-	Rif +
M3	control-evolved	MS2	-	Rif -
M4	control-evolved	MR2	-	Rif +
M5	control-evolved	MS3	-	Rif -
M6	control-evolved	MR3	-	Rif +

Supplementary Table 2. List of primers used for Multiplex FreqSeq. Right-side and left-sided barcodes are emboldened.

**Name**		**Sequence** (5'- 3')
For bridging primer synthesis		
Forward primer ABC1		AATGATACGGCGACCAC
Reverse primer ABC2		ACTGGCCGTCGTTTTAC
For FreqSeq PCR reaction 1		
Forward primer for *E. coli* (M13f tail) + genomic region		(GTAAAACGACGGCCAGT) GCAAAAACGTGGCGTATGTA
Reverse primer for *E. coli* (P7) + genomic region		(GATCGGAAGAGCACACGTCTGAACTCCAGTCA) TGACCTTCACCACCGATGTA
Forward primer for *M. xanthus* (M13f tail) + genomic region		(GTAAAACGACGGCCAGT) AGACGAACCCCCTGTCCGAA
Reverse primer for *M. xanthus* (reverse complement of P7) + genomic region		(TGACTGGAGTTCAGACGTGTGCTCTTCCGATC) ATGGGGCAGATGCGGCCGTA
For FreqSeq PCR reaction 2		
Forward primer ABC1		AATGATACGGCGACCAC
Bridging primer*		(AATGATACGGCGACCACCGAGATCTACACTCTTTCCCTACAC GACGCTCTTCCGATCT) (**NNNNNN**) (GTAAAACGACGGCCAGT)
Reverse primer with left-sided barcode<br><br>(Illumina-B adapter) + (**barcode**) + (P7) for *E. coli*	BCe_01	(CAAGCAGAAGACGGCATACGAGAT) (**AGCAAT**) (GATCGGAAGAGCACACG)
	BCe_02	(CAAGCAGAAGACGGCATACGAGAT) (**CCTGTT**) (GATCGGAAGAGCACACG)
	BCe_03	(CAAGCAGAAGACGGCATACGAGAT) (**GGGTTT**) (GATCGGAAGAGCACACG)
	BCe_04	(CAAGCAGAAGACGGCATACGAGAT) (**GAAGGC**) (GATCGGAAGAGCACACG)
	BCe_46	(CAAGCAGAAGACGGCATACGAGAT) (**GGATTA**) (GATCGGAAGAGCACACG)
	BCe_47	(CAAGCAGAAGACGGCATACGAGAT) (**TATATA**) (GATCGGAAGAGCACACG)
	BCe_48	(CAAGCAGAAGACGGCATACGAGAT) (**GTACAA**) (GATCGGAAGAGCACACG)
Reverse primer with left-sided barcode<br><br>(Illumina-B adapter) + (**barcode**) + (P7) for *M. xanthus*	BCm_01	(CAAGCAGAAGACGGCATACGAGAT) (**AGCAAT**) (TGACTGGAGTTCAGACG)
	BCm_02	(CAAGCAGAAGACGGCATACGAGAT) (**CCTGTT**) (TGACTGGAGTTCAGACG)
	BCm_03	(CAAGCAGAAGACGGCATACGAGAT) (**GGGTTT**) (TGACTGGAGTTCAGACG)
	BCm_46	(CAAGCAGAAGACGGCATACGAGAT) (**GGATTA**) (TGACTGGAGTTCAGACG)
	BCm_47	(CAAGCAGAAGACGGCATACGAGAT) (**TATATA**) (TGACTGGAGTTCAGACG)
	BCm_48	9CAAGCAGAAGACGGCATACGAGAT) (**GTACAA**) (TGACTGGAGTTCAGACG)

*NNNNNN denotes the 6-nt barcode and is the same as those used by Chubiz *et al* (2012)[1].

Supplementary Table 3. PCR conditions for Multiplex FreqSeq.

FreqSeq	DNA template	PCR mix	Cycling conditions
PCR1	Lysate of whole populations	2.5 µL Forward primer<br>2.5 µL Reverse primer<br>25 µL Phusion master mix<br>1.5 µL DMSO<br>8.0 µL DNA<br>10.5 µL water (to make a final volume of 50 µl)	98 °C – 2 mins<br>Cycle 15x:<br>98 °C – 10 s<br>56 °C (prey) or 57 °C (predator) – 30 s<br>72 °C – 30 s<br>72 °C – 7 mins<br>4 °C – <3 hrs or freeze
PCR2	Ampure XP bead purified product from PCR1	2.5 µL Forward primer ABC1<br>2.5 µL Reverse primer with left-sided barcode<br>25 µL Phusion master mix<br>1.5 µL DMSO<br>18.5 µL DNA<br>0.25 µM Bridging primer (volume to be determined according the DNA concentration of each primer)	98 °C – 3 mins<br>Cycle 15x:<br>98 °C – 20 s<br>53 °C – 20 s<br>72 °C – 30 s<br>72 °C – 7 mins<br>4 °C – <3 hrs or freeze

Supplementary Table 4. Mucoid frequencies. Frequencies of mucoid *E. coli* clones at the end of cycle 18 in each coevolved (ME1 to ME12) and control-evolved (E1 to E6) population. Values indicate mean frequencies from at least three independent samples ± 95% confidence intervals.

Population	Mucoid frequency
ME1	0.0008 ± 0.002
ME2	0
ME3	0.026 ± 0.025
ME4	0.203 ± 0.013
ME5	0.041 ± 0.044
ME6	0.043 ± 0.038
ME7	0.008 ± 0.003
ME8	0.138 ± 0.030
ME9	0.006 ± 0.012
ME10	0
ME11	0.147 ± 0.021
ME12	0.165 ± 0.049
E1	0
E2	0
E3	0
E4	0
E5	0
E6	0.0052 ± 0.01

Supplementary Table 5. Prey mutations. Accumulated mutations across three clones isolated from each control-evolved (E1 to E6) and coevolved (ME1 to ME12) prey population. Multiple mutations listed for the same gene in the same population correspond to different clones. Prey clones from ME1 are hyper-mutators and are not included in this table.

Pop.	Position	Gene	Mutation	Annotation	In clone(s)
E01	1,979,391	*insA*/*uspC*	IS*1* +8 bp	intergenic (-176/-355)	1
	3,815,810	*pyrE*/*rph*	Δ1 bp	intergenic (-42/+24)	1
	4,182,820	*rpoB* →	C→T	H526Y (CAC→TAC)	2
E02	1,712,582	*nth*/*dtpA*	C→T	intergenic (+424/-187)	2
	3,815,810	*pyrE*/*rph*	Δ1 bp	intergenic (-42/+24)	2
	3,815,859	*rph* ←	Δ82 bp	pseudogene (610-691/716 nt)	1, 3
	4,608,449	*rimI* →	A→C	I89L (ATC→CTC)	3
E03	1,979,328	*insA*/*uspC*	IS*1* +9 bp	intergenic (-113/-417)	2
E04	3,938,971	*rbsR* →	A→T	M249L (ATG→TTG)	1
	3,815,883	*rph* ←	+C	pseudogene (667/716 nt)	1, 2, 3
	4,353,011	*ghoT* →	IS*5* +4 bp	coding (104-107/174 nt)	2, 3
E05	2,773,416	*yfjW* →	$(T)_{8\to7}$	coding (99/1704 nt)	2
E06	3,213,316	*rpoD* →	A→C	E90D (GAA→GAC)	2
	3,815,859	*rph* ←	Δ82 bp	pseudogene (610-691/716 nt)	1, 2, 3
ME1	4,397,624	*mutL*	$(GCTGGC)_{3\to2}$	coding (213-218/1848 nt)	1, 2, 3
	20, 22 and 14 additional mutations in clones 1, 2 and 3 respectively. Detailed lists available upon request.				
ME2	584,808	*ompT* ←	IS*1* +8 bp	coding (819-826/954 nt)	3
	585,189	*ompT* ←	C→A	G149* (GGA→TGA)	2
	676,343	*ybeQ* ←	IS*5* +4 bp	coding (202-205/978 nt)	1
	1,879,829	*yeaR* ←	IS*186* +6 bp	coding (115-120/360 nt)	3
	3,815,859	*rph* ←	Δ82 bp	pseudogene (610-691/716 nt)	3
	3,815,921	*rph* ←	Δ1 bp	pseudogene (629/716 nt)	1, 2
	3,938,937	*rbsR* →	IS*2* +5 bp	coding (711-715/993 nt)	1
ME3	575,786	*[nmpC]*–*[ybdG]*	Δ27,775 bp	IS*5*-mediated	2
	3,231,367	*ygjK*/*fadH*	A→G	intergenic (+128/-298)	1
	3,378,982	*zapE* ←	A→G	L339P (CTG→CCG)	2
	3,815,806	*pyrE*/*rph*	A→C	intergenic (-38/+28)	2
	3,815,859	*rph* ←	Δ82 bp	pseudogene (610-691/716 nt)	1, 3
ME4	458,790	*clpX*/*lon*	IS*186* +6 bp	intergenic (+90/-93)	3
	585,015	*ompT* ←	IS*3* +4 bp	coding (616-619/954 nt)	1
	585,064	*ompT* ←	IS*3* +3 bp	coding (568-570/954 nt)	3
	1,188,482	*phoQ* ←	C→G	G432A (GGT→GCT)	3
	1,784,664	*fadK* →	IS*1* +9 bp	coding (1634-1642/1647 nt)	1

	3,815,859	*rph* ←	Δ82 bp	pseudogene (610-691/716 nt)	1, 2
	3,938,725	*rbsR* →	IS*5* +4 bp	coding (499-502/993 nt)	3
	4,237,542	*yjbG* →	T→G	Y216D (TAT→GAT)	3
	4,640,098	*arcA* ←	Δ1 bp	coding (209/717 nt)	3
ME5	1,405,766	*abgR*/*smrA*	G→A	intergenic (+117/-213)	1
	3,815,821	*pyrE*/*rph*	Δ13 bp	intergenic (-53/+1)	3
	3,815,859	*rph* ←	Δ82 bp	pseudogene (610-691/716 nt)	1, 2
ME6	585,171	*ompT* ←	IS*3* +3 bp	coding (461-463/954 nt)	1
	1,093,512	*ycdT* →	T→G	F213V (TTT→GTT)	3
	1,175,203	*ycfT*/*lolC*	A→T	intergenic (-38/-224)	3
	1,879,829	*yeaR* ←	IS*186* +6 bp	coding (115-120/360 nt)	3
	2,023,656	*fliR* →	IS*5* +4 bp	coding (764-767/786 nt)	2, 3
	3,815,821	*pyrE*/*rph*	Δ13 bp	intergenic (-53/+1)	1, 2, 3
	4,236,362	*yjbF* →	IS*1* +9 bp	coding (101-109/639 nt)	2
	4,400,484	*hfq* →	G→C	R66P (CGC→CCC)	3
ME7	585,273	*ompT* ←	IS*3* +3 bp	coding (359-361/954 nt)	1, 2
	1,979,337	*insA*/*uspC*	IS*5* +4 bp	intergenic (-122/-413)	3
	2,002,297	*fliC* ←	G→A	S437F (TCC→TTC)	3
	3,334,767	*ispB*/*sfsB*	$(A)_{5\rightarrow4}$	intergenic (+86/-142)	2
	3,815,890	*rph* ←	+C	pseudogene (660/716 nt)	1, 2, 3
ME8	575,786	*[nmpC]–[nfrA]*	Δ13,232 bp	IS*5*-mediated	2
	1,274,489	*ychO* →	C→A	R236S (CGC→AGC)	2
	2,023,826	*fliR*/*rcsA*	Δ1 bp	intergenic (+148/-142)	3
	3,461,564	*gspH* →	G→A	E33K (GAG→AAG)	1
	3,815,859	*rph* ←	Δ82 bp	pseudogene (610-691/716 nt)	1, 3
	3,848,559	*uhpC* ←	G→A	A22A (GCC→GCT)	2
	4,235,895	*pgi* → / → *yjbE*	IS*1* +9 bp	intergenic (+488/-3)	3
ME9	566,524	*[peaD]–[cusC]*	Δ30,005 bp	43 genes	1, 2, 3
	1,299,438	*ychE*/*oppA*	G→T	intergenic (+193/-1744)	1, 2, 3
	3,815,859	*rph* ←	Δ82 bp	pseudogene (610-691/716 nt)	1, 2, 3
	3,939,028	*rbsR* →	IS*2* +5 bp	coding (802-806/993 nt)	1, 2, 3
ME10	575,786	*[nmpC]–[nfrA]*	Δ14,013 bp	IS*5*-mediated	1
	585,064	*ompT* ←	IS*3* +3 bp	coding (568-570/954 nt)	3
	3,815,859	*rph* ←	Δ82 bp	pseudogene (610-691/716 nt)	1, 2
	3,853,838	*tisB*/*emrD*	C→T	intergenic (+196/-84)	2
	3,938,786	*rbsR* →	Δ20 bp	coding (560-579/993 nt)	3
	3,939,062	*rbsR* →	IS*2* +5 bp	coding (836-840/993 nt)	1, 2
ME11	458,790	*clpX*/*lon*	IS*186* +6 bp	intergenic (+90/-93)	3

	575,786	*[nmpC]–[ompT]*	Δ9,723 bp	IS*5*-mediated	1, 2
	732,995	*rhsC* →	C→T	T1138M (ACG→ATG)	3
	3,334,251	*ispB* →	G→A	C181Y (TGT→TAT)	3
	3,815,859	*rph* ←	Δ82 bp	pseudogene (610-691/716 nt)	1, 2, 3
	4,238,031	*yjbH* →	C→T	T133M (ACG→ATG)	3
	4,556,002	*yjiC* ←	IS*5* +4 bp	coding (316-319/831 nt)	3
ME12	458,790	*clpX*/*lon*	IS*186* +6 bp	intergenic (+90/-93)	3
	585,673	*ompT*/*pauD*	A→C	intergenic (-40/-384)	1, 2
	1,299,432	*ychE*/*oppA*	G→T	intergenic (+187/-1750)	3
	1,583,303	*ydeO* ←	IS*5* +4 bp	coding (382-385/762 nt)	1
	2,176,279	*gatZ* ←	IS*1* +9 bp	coding (35-43/1263 nt)	3
	2,565,938	*eutA* ←	C→T	G316D (GGC→GAC)	3
	3,815,810	*pyrE*/*rph*	Δ1 bp	intergenic (-42/+24)	1, 2
	3,938,996	*rbsR* →	T→A	L257* (TTA→TAA)	3
	4,238,888	*yjbH* →	IS*1* +8 bp	coding (1255-1262/2097 nt)	3
	4,640,418	*yjjY* →	IS*30* +2 bp	coding (17-18/141 nt)	3

Mutations and annotations follow standard breseq format. Flanking genes are mentioned for mutations in intergenic regions (e.g., *insA*/*uspC*). The nucleotide position corresponding to flanking genes is mentioned in brackets in the annotation column with + indicating position downstream of the stop codon and – indicating nucleotide upstream of the start codon of the following gene. SNPs are mentioned with an arrow and detail the corresponding amino-acid change for non-synonymous substitutions. Δ represents deletions, and for IS mediated mutations, corresponding IS element is mentioned as per reference-genome annotation. Large deletions mention first and last gene in the deleted segment in square brackets (e.g., *[nmpC]–[ybdG])*. * indicates change to stop codon.

Supplementary Table 6. Predator mutations. Accumulated mutations across three clones from each control-evolved (M2 to M4) and coevolved (ME1 to ME12) predator population. Multiple hits on the same gene in the same population correspond to different clones. All three predator clones from ME4 and two clones from ME8 are hypermutators and are not included in this table.

Pop.	Position	Gene	Mutation	Annotation	In clone(s)
M2	335,653	*MXAN_RS01385*	C→A	Y55* (TAC→TAA)	2
	1,837,263	*MXAN_RS07605*	G→T	L31F (TTG→TTT)	3
	3,600,700	*MXAN_RS14915*	A→T	H548L (CAC→CTC)	2,3
	6,885,348	*MXAN_RS26830/ MXAN_RS26835*	A→G	intergenic (+1934/+1898)	3
	7,536,463	*MXAN_RS29545*	T→G	S142R (AGC→CGC)	3
M3	2,634,706	*MXAN_RS10970*	C→T	G630D (GGC→GAC)	1,2,3
	7,266,486	*MXAN_RS28390*	T→G	Y262D (TAC→GAC)	1,2,3
M4	1,837,910	*MXAN_RS07605*	G→T	S247I (AGC→ATC)	2
	2,104,526	*MXAN_RS08605*	C→T	P39S (CCC→TCC)	1
	3,600,700	*MXAN_RS14915*	A→T	H548L (CAC→CTC)	1,2,3
	5,275,860	*MXAN_RS20850*	G→T	A980E (GCG→GAG)	1
	7,266,516	*MXAN_RS28390*	G→T	G272C (GGC→TGC)	1
ME1	846,285	*MXAN_RS03570*	A→G	R12R (CGA→CGG)	1
	3,110,383	*MXAN_RS12885*	G→A	G449R (GGG→AGG)	1
	7,128,080	*MXAN_RS27920*	G→C	G59A (GGC→GCC)	1
ME2	3,600,700	*MXAN_RS14915*	A→T	H548L (CAC→CTC)	1,2,3
	4,835,633	*MXAN_RS19305*	C→T	A154T (GCG→ACG)	1,2
	5,245,952	*MXAN_RS20780*	G→C	G431G (GGC→GGG)	1
	6,783,715	*MXAN_RS26430*	A→C	D483A (GAC→GCC)	1
	7,128,607	*MXAN_RS27920*	A→C	T235P (ACG→CCG)	3
	7,128,784	*MXAN_RS27920*	G→T	E294* (GAG→TAG)	1,2
	7,405,346	*MXAN_RS29015*	C→T	Q56* (CAG→TAG)	1
ME3	1,099,133	*MXAN_RS04600*	C→A	T325T (ACC→ACA)	1,2,3
	3,103,710	*MXAN_RS12865 / MXAN_RS12870*	C→T	intergenic (-17/+70)	1,2,3
	7,128,379	*MXAN_RS27920*	G→A	A159T (GCG→ACG)	2
	7,128,860	*MXAN_RS27920*	$(CTGAA)_{1\rightarrow 2}$	coding (956/1269 nt)	1,3
ME4	4,686,452	*mutS*	T→G	S665R (AGC→CGC)	1, 2, 3
	6,661,644	*recN*	T→C	N283S (AAC→AGC)	1, 2, 3

94, 64 and 107 additional mutations in clones 1, 2 and 3 respectively. Detailed lists available upon request. Detailed lists available upon request.					
ME5	3,013,847	*MXAN_RS12525*	G→T	A98E (GCG→GAG)	1
	3,107,516	*MXAN_RS12875*	A→C	L207R (CTC→CGC)	3
	3,111,550	*MXAN_RS12890*	G→T	R325L (CGC→CTC)	1
	3,600,700	*MXAN_RS14915*	A→T	H548L (CAC→CTC)	2
	4,990,717	*MXAN_RS19805*	C→T	L138L (CTG→CTA)	2
	6,868,901	*MXAN_RS26750*	C→A	A92E (GCG→GAG)	1,3
	7,127,967	*MXAN_RS27920*	T→A	Y21* (TAT→TAA)	2
	7,128,926	*MXAN_RS27920*	C→G	P341R (CCC→CGC)	3
	7,128,928	*MXAN_RS27920*	A→T	I342F (ATC→TTC)	3
	7,128,968	*MXAN_RS27920*	G→T	R355L (CGA→CTA)	1
ME6	3,600,700	*MXAN_RS14915*	A→T	H548L (CAC→CTC)	1,2,3
	7,128,205	*MXAN_RS27920*	G→C	G101R (GGG→CGG)	2
	7,128,992	*MXAN_RS27920*	T→A	V363E (GTG→GAG)	1,3
ME7	190,820	*MXAN_RS00760* / *MXAN_RS00765*	$(C)_{7\rightarrow6}$	intergenic (-221/-160)	2
	447,814	*MXAN_RS01880*	T→C	F197S (TTC→TCC)	2
	1,923,559	*MXAN_RS07875*	G→T	T792T (ACG→ACT)	1
	3,013,847	*MXAN_RS12525*	G→T	A98E (GCG→GAG)	1,3
	3,600,700	*MXAN_RS14915*	A→T	H548L (CAC→CTC)	1,2,3
	4,779,681	*MXAN_RS19125*	T→G	S1372R (AGC→CGC)	2
	7,128,142	*MXAN_RS27920*	G→A	G80R (GGG→AGG)	1,3
	7,128,944	*MXAN_RS27920*	T→A	V347D (GTC→GAC)	2
	8,557,472	*MXAN_RS33865*	C→T	L700L (CTG→CTA)	2
ME8	4,688,236	*mutS*	A→G	F70S (TTC→TCC)	1, 2
42 and 44 additional mutations in clones 1 and 2 respectively. Detailed lists available upon request.					
ME8	1,202,985	*MXAN_RS04945*	+TTC	coding (666/1143 nt)	3
	7,127,965	*MXAN_RS27920*	Δ1 bp	coding (61/1269 nt)	3
	8,560,499	*MXAN_RS33870* / *MXAN_RS33875*	T→C	intergenic(-91/-340)	3
ME9	3,107,516	*MXAN_RS12875*	A→C	L207R(CTC→CGC)	3
	4,780,726	*MXAN_RS19125*	$(C)_{8\rightarrow7}$	coding(3069/26949nt)	1
	6,868,901	*MXAN_RS26750*	C→A	A92E(GCG→GAG)	3
	7,125,516	*[MXAN_RS27905]-[MXAN_RS27930]*	Δ5,485bp		2
	7,128,926	*MXAN_RS27920*	C→G	P341R(CCC→CGC)	3
	7,128,928	*MXAN_RS27920*	A→T	I342F(ATC→TTC)	3
	7,129,049	*MXAN_RS27920*	C→T	A382V(GCG→GTG)	1
	7,133,777	*MXAN_RS27935*	T→G	S120R(AGC→CGC)	2

	8,065,362	*MXAN_RS31715*	G→T	E669*(GAA→TAA)	2
ME10	865,277	*MXAN_RS03650*	C→T	T270T(ACG→ACA)	1
	1,099,133	*MXAN_RS04600*	C→A	T325T(ACC→ACA)	1,2,3
	3,103,710	*MXAN_RS12865* / *MXAN_RS12870*	C→T	intergenic(-17/+70)	1,2,3
	7,129,100	*MXAN_RS27920*	T→G	L399R(CTC→CGC)	1,2,3
ME11	1,295,240	*MXAN_RS05350*	G→A	L33L(CTG→TTG)	3
	3,013,847	*MXAN_RS12525*	G→T	A98E(GCG→GAG)	3
	3,600,700	*MXAN_RS14915*	A→T	H548L(CAC→CTC)	1,2,3
	3,757,170	*MXAN_RS15525*	G→C	N62K(AAC→AAG)	1,3
	7,128,515	*MXAN_RS27920*	A→C	D204A(GAT→GCT)	1,3
	7,129,015	*MXAN_RS27920*	G→A	D371N(GAC→AAC)	2
ME12	1,055,109	*MXAN_RS04445*	A→C	D244A(GAC→GCC)	3
	3,013,847	*MXAN_RS12525*	G→T	A98E(GCG→GAG)	3
	3,108,002	*MXAN_RS12875*	G→A	S45L(TCG→TTG)	2
	6,696,636	*MXAN_RS26090* / *MXAN_RS26095*	T→C	intergenic(-172/-68)	1
	6,868,901	*MXAN_RS26750*	C→A	A92E(GCG→GAG)	1,2,3
	6,925,823	*MXAN_RS27010*	G→A	P119L(CCG→CTG)	2
	6,958,933	*MXAN_RS27125*	C→T	A557T(GCG→ACG)	1
	7,128,926	*MXAN_RS27920*	C→G	P341R(CCC→CGC)	1,2
	7,128,928	*MXAN_RS27920*	A→T	I342F(ATC→TTC)	1,2
	7,129,072	*MXAN_RS27920*	G→A	A390T(GCC→ACC)	3

Supplementary Table 7. Genes mutated in prey. Mutations in genes or upstream of genes in *E. coli* from the evolution experiment. Numbers and brackets indicate in how many clones and populations, respectively, the gene was mutated. Mutations from *E. coli* from ME1 and synonymous SNPs are not included in the table. Mutations in rows 2-5 are big deletions for which the first and last gene are mentioned (all four deletions encompass *ompT*).

Gene(s) or upstream of gene	Control (#)	Coevolved (#)	Total (#)
clpX	0	3(3)	3(3)
[peaD]–[cusC]	0	3(1)	3(1)
[nmpC]–[ompT]	0	2(1)	2(1)
[nmpC]–[nfrA]	0	2(2)	2(2)
[nmpC]–[ybdG]	0	1	1
ompT	0	11(6)	11(6)
ybeQ	0	1	1
rhsC	0	1	1
ycdT	0	1	1
ycfT	0	1	1
phoQ	0	1	1
ychO	0	1	1
oppA	0	4(2)	4(2)
smrA	0	1	1
ydeO	0	1	1
dtpA	1	0	1
fadK	0	1	2
yeaR	0	2(2)	2(2)
insA / *uspC**	2(2)	1	3(3)
fliC	0	1	1
fliR	0	2(2)	2(2)
rcsA	0	1	1
gatZ	0	1	1
eutA	0	1	1
yfjW	1	0	1
rpoD	1	0	1
fadH	0	1	1
ispB	0	1	1
sfsB	0	1	1
zapE	0	1	1
gspH	0	1	1
rph	10(4)	29(11)	39(15)

emrD	0	1	1
rbsR	1	10(6)	11(7)
rpoB	1	0	1
*yjbEFGH***	0	5(4)	5(4)
ghoT	2(1)	0	2(1)
hfq	0	1	1
yjiC	0	1	1
rimI	1	0	1
arcA	0	1	1
yjjY	0	1	1

*Genes face each other and mutation is seen in the intergenic region, thus upstream of both genes

**Functional operon involved in polysaccharide transport

Supplementary Table 8. Genes mutated in predator. Mutations in genes or upstream of genes in *M. xanthus* from the evolution experiment. Numbers and brackets indicate in how many clones and populations, respectively, the gene was mutated. Mutations from all three ME4 predator clones, two ME8 clones and synonymous SNPs are not included in the table.

Gene(s) or upstream of gene	Control (#)	Coevolved (#)	Total (#)
MXAN_RS00760 / MXAN_RS00765	0	1(1)	1(1)
MXAN_RS01385	1(1)	0	1(1)
MXAN_RS01880	0	1(1)	1(1)
MXAN_RS03570	0	1(1)	1(1)
MXAN_RS03650	0	1(1)	1(1)
MXAN_RS04445	0	1(1)	1(1)
MXAN_RS04600	0	6(2)	6(2)
MXAN_RS05350	0	1(1)	1(1)
MXAN_RS07605	2(2)	0	2(2)
MXAN_RS07875	0	1(1)	1(1)
MXAN_RS08605	1(1)	0	1(1)
MXAN_RS10970	3(1)	0	3(1)
MXAN_RS12525	0	5(4)	5(4)
MXAN_RS12865	0	6(2)	6(2)
MXAN_RS12875	0	4(4)	4(4)
MXAN_RS12885	0	1(1)	1(1)
MXAN_RS12890	0	1(1)	1(1)
MXAN_RS14915	8(3)	15(6)	23(9)
MXAN_RS15525	0	2(1)	2(1)
MXAN_RS17875	0	1(1)	1(1)
MXAN_RS19125	0	2(2)	2(2)
MXAN_RS19305	0	2(1)	2(1)
MXAN_RS19805	0	1(1)	1(1)
MXAN_RS20780	0	1(1)	1(1)
MXAN_RS20850	1(1)	0	1(1)
MXAN_RS26090 / MXAN_RS26095	0	1(1)	1(1)
MXAN_RS26430	0	1(1)	1(1)
MXAN_RS26750	0	6(3)	6(3)
MXAN_RS26830 / MXAN_RS26835	1(1)	0	1(1)
MXAN_RS27010	0	1(1)	1(1)
MXAN_RS27125	0	1(1)	1(1)
[MXAN_RS27905]–[MXAN_RS27930]	0	1(1)	1(1)
MXAN_RS27920	0	28(12)	28(12)
MXAN_RS27935	0	1(1)	1(1)
MXAN_RS28390	4(2)	0	4(2)
MXAN_RS29015	0	1(1)	1(1)
MXAN_RS29545	1(1)	0	1(1)
MXAN_RS31715	0	1(1)	1(1)
MXAN_RS33865	0	1(1)	1(1)

Supplementary Table 9. Distribution of *Mxan_RS27920* homologs. All homologs detected using the blastn[2] suite against a database of all RefSeq representative genomes are shown.

Species	Score (bits)	E value	Identity	Accession
Myxococcus xanthus DK 1622	2344	0	100%	NC_008095.1
Myxococcus macrosporus strain HW-1	1989	0	95.2%	NC_015711.1
Myxococcus stipitatus DSM 14675	1312	0	86.5%	NC_020126.1
Corallococcus coralloides DSM 2259	1182	0	84.7%	NC_017030.1
Hyalangium minutum strain DSM 14724	944	0	81.6%	NZ_JMCB01000017.1
Stigmatella aurantiaca DW4/3-1	817	0	79.2%	NC_014623.1
Archangium gephyra strain DSM 2261	761	0	78.8%	NZ_CP011509.1
Cystobacter fuscus DSM 2262	725	0	78.2%	NZ_ANAH02000006.1
Vulgatibacter incomptus strain DSM 27710	67.6	1.00E-07	90.2%	NZ_CP012332.1

Supplementary Note 1: Bash script used for Multiplex Freqseq

```
#Trims the Illumina adapter from the 3' end exposing the 6-nt barcode. Create adapter.fa file beforehand with the adapter sequence.
java -jar ~/trimmomatic-0.33.jar SE 1_S1_L001_R1_001.fastq.gz trimmed.fastq.gz ILLUMINACLIP:adapter.fa:2:30:10

#unzips the fastq file to be used by FASTX toolkit
gunzip trimmed.fastq.gz

#Create barcodes.txt file beforehand with all the right-side barcodes used
cat trimmed.fastq | fastx_barcode_splitter.pl --bcfile barcodes.txt --eol --mismatches 1 --prefix

#Demultiplexes the sequences as per the right-side barcodes
~/bla_ --suffix ".fastq"


#####Start here if demultiplexing has been done by the MiSeq machine#########

#invokes Freq-Out to determine ratio of each allele for each barcode
mono freqout.exe -xml=settings_rpsL.xml bla_BC1.fastq

#copies and renames the output from Freq-Out so that subsequent commands do not overwrite the previous output file
cp AF_Seq_Results.csv BC_01.csv

#repeat for each right-side barcode
mono freqout.exe -xml=settings_rpsL.xml bla_BC2.fastq
cp AF_Seq_Results.csv BC_02.csv
mono freqout.exe -xml=settings_rpsL.xml bla_BC3.fastq
cp AF_Seq_Results.csv BC_03.csv
mono freqout.exe -xml=settings_rpsL.xml bla_BC4.fastq
cp AF_Seq_Results.csv BC_04.csv
mono freqout.exe -xml=settings_rpsL.xml bla_BC46.fastq
cp AF_Seq_Results.csv BC_46.csv
mono freqout.exe -xml=settings_rpsL.xml bla_BC47.fastq
cp AF_Seq_Results.csv BC_47.csv
mono freqout.exe -xml=settings_rpsL.xml bla_BC48.fastq
cp AF_Seq_Results.csv BC_48.csv

#removes the original copy of last generated output file
rm AF_Seq_Results.csv
```

Supplementary References

1. Chubiz, L. M., Lee, M.-C., Delaney, N. F. & Marx, C. J. FREQ-Seq: A Rapid, Cost-Effective, Sequencing-Based Method to Determine Allele Frequencies Directly from Mixed Populations. *PLoS One* **7**, e47959 (2012).

2. Zhang, Z., Schwartz, S., Wagner, L. & Miller, W. A Greedy Algorithm for Aligning DNA Sequences. *J. Comput. Biol.* (2000). doi:10.1089/10665270050081478
