# Supplementary material for: Bacterial predator-prey coevolution accelerates genome evolution and selects on virulence-associated prey defences
Source: Nat Commun. 2019 Sep 20;10:4301. doi: 10.1038/s41467-019-12140-6 (PMC6754418; doi:10.1038/s41467-019-12140-6)
Supplement: Supplementary file 2 — Reporting Summary [file 41467_2019_12140_MOESM2_ESM.pdf]

nature research

Corresponding author(s): Ramith R. Nair
Marie Vasse

Reporting Summary

Nature Research wishes to improve the reproducibility of the work that we publish. This form provides structure for consistency and transparency in reporting. For further information on Nature Research policies, see Authors & Referees and the Editorial Policy Checklist.

Statistical parameters

When statistical analyses are reported, confirm that the following items are present in the relevant location (e.g. figure legend, table legend, main text, or Methods section).

n/a	Confirmed	
☐	☒	The exact sample size (n) for each experimental group/condition, given as a discrete number and unit of measurement
☐	☒	An indication of whether measurements were taken from distinct samples or whether the same sample was measured repeatedly
☐	☒	The statistical test(s) used AND whether they are one- or two-sided<br>*Only common tests should be described solely by name; describe more complex techniques in the Methods section.*
☐	☒	A description of all covariates tested
☐	☒	A description of any assumptions or corrections, such as tests of normality and adjustment for multiple comparisons
☐	☒	A full description of the statistics including central tendency (e.g. means) or other basic estimates (e.g. regression coefficient) AND variation (e.g. standard deviation) or associated estimates of uncertainty (e.g. confidence intervals)
☐	☒	For null hypothesis testing, the test statistic (e.g. F, t, r) with confidence intervals, effect sizes, degrees of freedom and P value noted<br>*Give P values as exact values whenever suitable.*
☒	☐	For Bayesian analysis, information on the choice of priors and Markov chain Monte Carlo settings
☒	☐	For hierarchical and complex designs, identification of the appropriate level for tests and full reporting of outcomes
☒	☐	Estimates of effect sizes (e.g. Cohen's d, Pearson's r), indicating how they were calculated
☐	☒	Clearly defined error bars<br>*State explicitly what error bars represent (e.g. SD, SE, CI)*

Our web collection on statistics for biologists may be useful.

Software and code

Policy information about availability of computer code

Data collection	breseq v0.27.0, SPAdes v3.11.1, FastQC, Trimmomatic v0.33 and samtools v1.3.1 were used for whole genome analysis<br>FreqOut and FASTX-Toolkit were used for FreqSeq data analysis
Data analysis	The statistical analysis was performed using R studio (version 1.0.136) and the packages car and ggplot.

For manuscripts utilizing custom algorithms or software that are central to the research but not yet described in published literature, software must be made available to editors/reviewers upon request. We strongly encourage code deposition in a community repository (e.g. GitHub). See the Nature Research guidelines for submitting code & software for further information.

Data

Policy information about availability of data

All manuscripts must include a data availability statement. This statement should provide the following information, where applicable:

- Accession codes, unique identifiers, or web links for publicly available datasets
- A list of figures that have associated raw data
- A description of any restrictions on data availability

Additional data is either provided or had been uploaded to GenBank

Field-specific reporting

Please select the best fit for your research. If you are not sure, read the appropriate sections before making your selection.

☒ Life sciences ☐ Behavioural & social sciences ☐ Ecological, evolutionary & environmental sciences

For a reference copy of the document with all sections, see nature.com/authors/policies/ReportingSummary-flat.pdf

Life sciences study design

All studies must disclose on these points even when the disclosure is negative.

Sample size	Antibiotic resistant and sensitive predator and prey were mixed in all possible combinations (total of 4). We maintained three independent replicates for each combination to give 12 independent replicate lineages.
Data exclusions	We excluded the Illumina data for which less than 10000 reads per barcode combination were available to make sure we use only reliable data. In addition, we excluded the Illumina data that corresponded to ratios of competitors lower than 5% as this was outside of our validity range. All these exclusion criteria were decided before running the experiments.
Replication	We performed at least 3 replicates per experiment and all attempts at replication were successful. Replication details for each experiment is mentioned in the manuscript.
Randomization	The experiments took place in individual flasks. As a consequence, randomization is not relevant to our study.
Blinding	All the samples within a replicate were treated together and equally so blinding was not relevant to our study.

Reporting for specific materials, systems and methods

Materials & experimental systems

n/a	Involved in the study
☒	☐ Unique biological materials
☒	☐ Antibodies
☒	☐ Eukaryotic cell lines
☒	☐ Palaeontology
☒	☐ Animals and other organisms
☒	☐ Human research participants

Methods

n/a	Involved in the study
☒	☐ ChIP-seq
☒	☐ Flow cytometry
☒	☐ MRI-based neuroimaging
